# World Heart Federation Cholesterol Roadmap 2022

**DOI:** 10.5334/gh.1154

**Published:** 2022-10-14

**Authors:** Kausik K. Ray, Brian A. Ference, Tania Séverin, Dirk Blom, Stephen J. Nicholls, Mariko H. Shiba, Wael Almahmeed, Rodrigo Alonso, Magdalena Daccord, Marat Ezhov, Rosa Fernández Olmo, Piotr Jankowski, Fernando Lanas, Roopa Mehta, Raman Puri, Nathan D. Wong, David Wood, Dong Zhao, Samuel S. Gidding, Salim S. Virani, Donald Lloyd-Jones, Fausto Pinto, Pablo Perel, Raul D. Santos

**Affiliations:** 1 Imperial Centre for Cardiovascular Disease Prevention, Department of Primary Care and Public Health, Imperial College London, Reynolds Building, St. Dunstans Road, London, GB; 2 Department of Public Health and Primary Care, British Heart Foundation, UK; 3 Cardiovascular Epidemiology Unit, Centre for Naturally Randomized Trials, University of Cambrige, Cambridge, GB; 4 World Heart Federation, Geneva, CH; 5 Department of Medicine, University of Cape Town, Cape Town, ZA; 6 Victorian Heart Institute, Monash University, Melbourne, AU; 7 Cardiovascular Center, Osaka Medical and Pharmaceutical University, Takatsuki, JP; 8 Cleveland Clinic Abu Dhabi, Abu Dhabi, AE; 9 Center for advanced metabolic medicine and nutrition, Santiago, CL; 10 Europe, Rochester, Kent, GB; 11 Chazov National Medical Research Center of Cardiology, Moscow, RW; 12 Cardiac Rehabilitation Unit Jaen University Hospital, Jean, ES; 13 Department of Internal Medicine and Geriatric Cardiology and Department of Epidemiology and Health Promotion, School of Public Health, Centre of Postgraduate Medical Education, Warsaw, PL; 14 Universidad de La frontera, Temuco, CL; 15 Instituto Nacional de Ciencias Medicas y Nutricion, Salvador Zubirán, Mexico City, MX; 16 Department of Cardiology, Apollo Hospital, New Delhi, IN; 17 University of California, Irvine, US; 18 Health, National University of Ireland Galway, Galway, IE; 19 Beijing Institute of Heart, Lung & Blood Vessel Diseases, Capital Medical University Beijing Anzhen Hospital, Beijing, CN; 20 Geisinger Genomic Medicine Institute, Danville, PS, US; 21 Baylor College of Medicine/Michael E. DeBakey Veterans Affairs Medical Center, Houston, TX, US; 22 Preventive medicine, Northwestern University, Chicago, US; 23 Lisbon School of Medicine, University of Lisbon, Lisbon, PT; 24 London School of Hygiene & Tropical Medicine and World Heart Federation, London, UK and Geneva, CH; 25 Cardiopneumology Department and Lipid Clinic, Heart Institute (InCor) University of Sao Paulo Medical School Hospital and Hospital Israelita Albert Einstein, Sao Paulo, BR

**Keywords:** cholesterol, low-density lipoprotein cholesterol prevention, ASCVD, lipid lowering therapy, familial hypercholesterolaemia

## Abstract

**Background:**

Atherosclerotic cardiovascular diseases (ASCVD) including myocardial infarction, stroke and peripheral arterial disease continue to be major causes of premature death, disability and healthcare expenditure globally. Preventing the accumulation of cholesterol-containing atherogenic lipoproteins in the vessel wall is central to any healthcare strategy to prevent ASCVD. Advances in current concepts about reducing cumulative exposure to apolipoprotein B (apo B) cholesterol-containing lipoproteins and the emergence of novel therapies provide new opportunities to better prevent ASCVD. The present update of the World Heart Federation Cholesterol Roadmap provides a conceptual framework for the development of national policies and health systems approaches, so that potential roadblocks to cholesterol management and thus ASCVD prevention can be overcome.

**Methods:**

Through a review of published guidelines and research papers since 2017, and consultation with a committee composed of experts in clinical management of dyslipidaemias and health systems research in low-and-middle income countries (LMICs), this Roadmap identifies (1) key principles to effective ASCVD prevention (2) gaps in implementation of these interventions (knowledge-practice gaps); (3) health system roadblocks to treatment of elevated cholesterol in LMICs; and (4) potential strategies for overcoming these.

**Results:**

Reducing the future burden of ASCVD will require diverse approaches throughout the life-course. These include: a greater focus on primordial prevention; availability of affordable cholesterol testing; availability of universal cholesterol screening for inherited dyslipidaemias; risk stratification moving beyond 10-year risk to look at lifetime risk with adequate risk estimators; wider availability of affordable cholesterol-lowering therapies which should include statins as essential medications globally; use of adequate doses of potent statin regimens; and combination therapies with ezetimibe or other therapies in order to attain and maintain robust reductions in LDL-C in those at highest risk. Continuing efforts are needed on health literacy for both the public and healthcare providers, utilising multi-disciplinary teams in healthcare and applications that quantify both ASCVD risk and benefits of treatment as well as increased adherence to therapies.

**Conclusions:**

The adverse effects of LDL-cholesterol and apo B containing lipoprotein exposure are cumulative and result in ASCVD. These are preventable by implementation of different strategies, aimed at efficiently tackling atherosclerosis at different stages throughout the human life-course. Preventive strategies should therefore be updated to implement health policy, lifestyle changes and when needed pharmacotherapies earlier with investment in, and a shift in focus towards, early preventive strategies that preserve cardiovascular health rather than treat the consequences of ASCVD.

## Introduction

**Figure 1:**
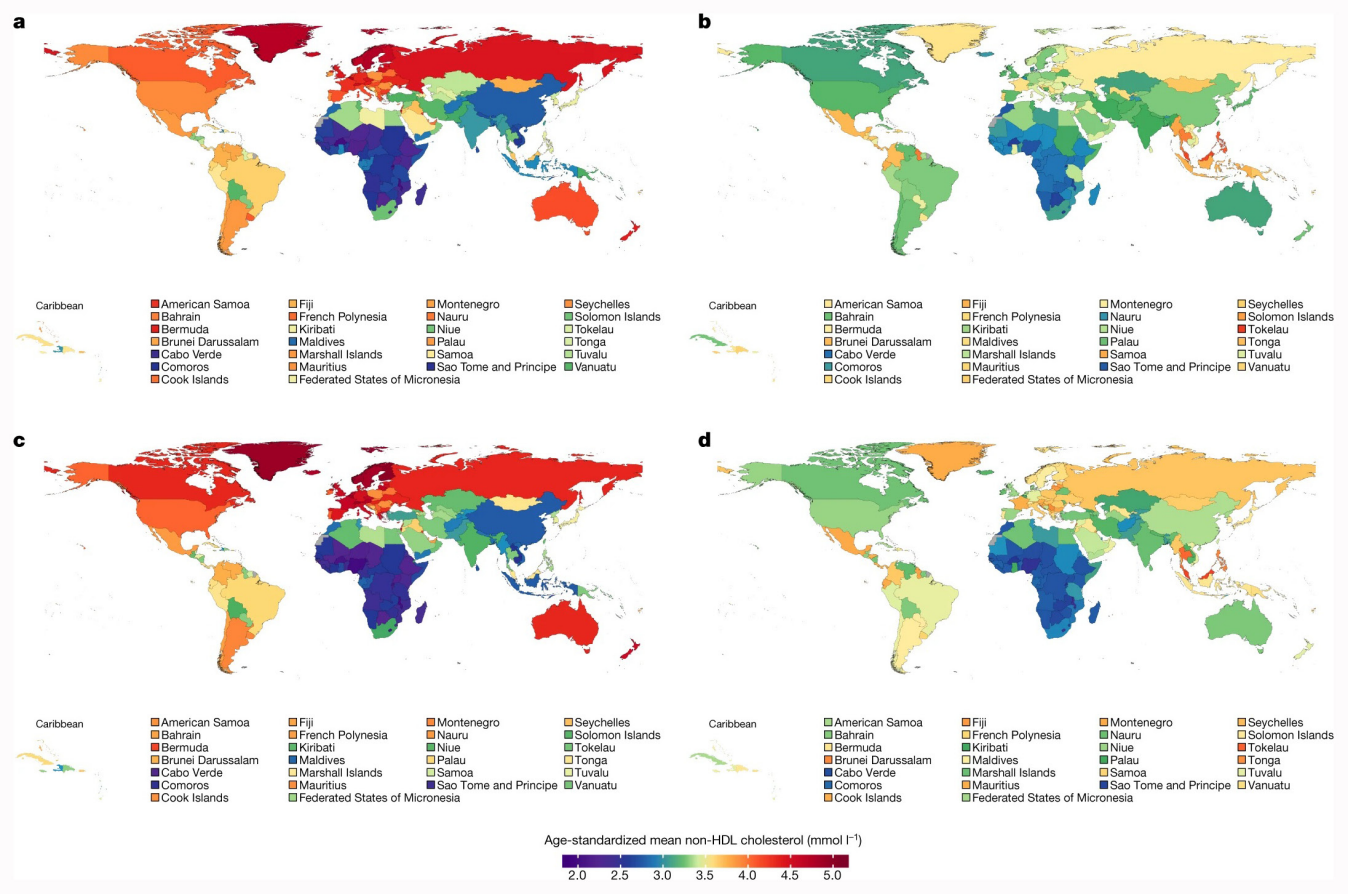
**Age-standardized mean non-HDL cholesterol (mmol/L^–1^) across the world from the NCD Risk Factor Collaboration.** Reproduced from NCD Risk Factor Collaboration (NCD-RisC). Repositioning of the global epicentre of non-optimal cholesterol. *Nature* **582**, 73–77 (2020). https://doi.org/10.1038/s41586-020-2338-1 licensed under a Creative Commons Attribution 4.0 International License, http://creativecommons.org/licenses/by/4.0/. **a**, Age-standardized mean non-HDL cholesterol in women in 1980. **b**, Age-standardized mean non-HDL cholesterol in women in 2018. **c**, Age-standardized mean non-HDL cholesterol in men in 1980. **d**, Age-standardized mean non-HDL cholesterol in men in 2018.

Atherosclerotic cardiovascular disease (ASCVD) remains the leading cause of death globally despite major advances in our understanding of the pathophysiology of atherosclerosis, its consequences and the development of new preventive therapies [1,2]. Of concern is the observation that ASCVD is increasing both in prevalence in low- and middle-income countries (LMICs), where the vast majority of the global population reside, but also presenting there a decade or more earlier than in higher income countries (Figure 1). When ASCVD impacts an individual’s life-course at such an early age, it not only has severe consequences for the person affected, but invariably impacts economic growth in affected countries, adversely affecting employability, productivity, and income potential during crucial years of the lifespan. Furthermore, adverse health of an individual impacts their caregivers and immediate family.

Different strategies are required to break the cycle of inequalities between wealth and health, resulting in adverse health outcomes and vice versa, which has been further compounded by the global coronavirus pandemic. Those most at risk for ASCVD because of obesity, hypertension, diabetes, and dyslipidaemia were particularly vulnerable to the virus. There has also been a loss of connection for many patients with their healthcare providers, leading to reduced screening, delay in diagnosis and loss of control of these chronic risk factors [3]. This will have ripple effects in ASCVD disease incidence for years to come.

In 2012, the World Health Organization (WHO) aimed to reduce premature mortality from non-communicable diseases (NCD) by 25% by the year 2025. This was soon followed by the United Nations Sustainable Development Goals (2015), which aimed to reduce premature mortality from NCD by 30% by 2030. A decade on these goals and the agenda for health and well-being are far from being realised with only 14 countries on track [4].

WHF Roadmaps provide a framework for public health policy, which build upon compelling scientific evidence, identify current roadblocks to implementation of best practice through surveys of WHF member countries and ultimately offer potential solutions to be adapted across all regions of the world over the next decade to suit local needs. The first WHF Cholesterol Roadmap was published in 2017 [5]. Since then, there have been advances in science, treatment, and technology. The present Roadmap update builds on the original publication and reviews:

- new evidence on the burden of disease, epidemiology, diagnosis, treatment, technologies and policies which can lead to improved outcomes for people living with inadequately controlled blood cholesterol and
- lessons learnt from the original WHF Cholesterol Roadmap implementation in different countries and settings.

This update was developed through a review of published guidelines and research papers, in consultation with a committee, composed of experts in clinical management of cholesterol, cardiovascular risk, health systems research and patient advocacy and support.

This present document is set out into three parts. The first section addresses fundamental concepts about lipids and ASCVD which are essential to inform solutions at health policy/funding level (macro-system) as well as guiding day to day interactions at the physician-patient level (micro-systems). This includes the role of high blood cholesterol levels in the global burden of disease (population approach) as distinct from the role of cholesterol lowering to mitigate ASCVD risk in those at greatest risk (high-risk approach); which lipid parameters to measure; how to assess those at higher risk of ASCVD; special populations with common inherited cholesterol disorders; and finally, pharmacotherapy. The second section provides case studies and examples of successful implementation strategies globally. Finally, the third section builds on a survey of member countries to inform current roadblocks and provides potential solutions to be implemented.

## Fundamental Concepts About Lipids and Ascvd – The 8 Pillars

There is irrefutable evidence for the causal role of LDL-cholesterol (LDL-C) in ASCVD. The writing committee recognises the impact of misinformation in social media, as well as conflicting data from dietary intervention trials, on the discussion about the role of blood cholesterol in ASCVD, which may serve as a barrier for better cardiovascular health and implementation of preventive strategies. In this regard several fundamental concepts have been established through multiple lines of scientific enquiry over many decades which are important to understand, and around which future implementation strategies can be built including improving physician and citizen health literacy and refuting misinformation. These “8 Pillars” are set out in **Box 1**.

**Box 1 The Fundamentable Principles of the Role of Lipids and Lipoproteins and Ascvd Risk and its Prevention – 8 Pillars**

1. Atherosclerosis results from the retention of apolipoprotein B (apo B) containing lipoproteins mostly in the form of low- density lipoproteins (LDL) in the vessel wall. LDL cholesterol (LDL-C) is not only causal but a cumulative risk factor, over the lifespan, for ASCVD.
2. Individuals have differential retention of apo B containing lipoproteins and hence differential vulnerability to the effects of LDL-C exposure. Therefore, LDL-C and apo B should not be considered in isolation without considering other factors.
3. Most cardiovascular events occur among individuals without extreme elevations in LDL-C, hence global risk should be considered.
4. Extreme elevations in LDL-C from birth with a monogenic basis (familial hypercholesterolaemia) are more common than previously recognised and are prevalent across all regions of the world, and their consequences are largely preventable through early screening and treatment.
5. ASCVD can be reduced through reductions in LDL-C through multiple different pathways, with benefit reliably quantifiable and proportional to both the absolute reduction in LDL-C and the duration of that reduction, hence treatments could be interchanged or combined as needed.
6. As the majority of the total atherogenic cholesterol content (non-HDL-C) consists of LDL-C and as the majority of apo B containing lipoproteins are LDL particles, LDL-C reductions will provide largely predictable parallel proportional reductions in non-HDL-C and apo B.
7. The increasing prevalence of cardio-metabolic diseases such as obesity and diabetes has resulted in an increase in other lipid disorders which increase ASCVD risk. These are characterised by elevated triglyceride rich apo B containing lipoproteins which are atherogenic. Therefore, pragmatically moving towards estimation of atherogenic particle number in the form of apo B measurements with a single measure, or if this is not feasible, total atherogenic cholesterol content (non-HDL-C) may improve risk assessment and measures of benefit irrespective of therapeutic modality.
8. The recognition that elevations in lipoprotein (a) are common (but poorly detected) and are an independent causal risk factor of ASCVD.

## The relevance of lipids and lipoproteins to the global burden of CVD

Worldwide, CVD affected 523 million individuals in 2019 [1]. It claims over 18 million lives each year, approximately 85% of which are due to ASCVD [1]. The Global Burden of Disease (GBD) Collaboration has demonstrated that assessment of total cholesterol alone is uninformative to assess time trends as it may mask opposing trends in HDL-C and non-HDL-C which have opposite associations with the risk of CVD [6,7,8]. The rise in obesity and diabetes globally results in dyslipidaemia characterised by a fall in HDL-C and a rise in non-HDL-C and high triglyceride levels [8,9], hence health systems should pragmatically move towards using non-HDL-C or apo B as a unifying measure of atherogenic lipid risk.

**Figure 2:**
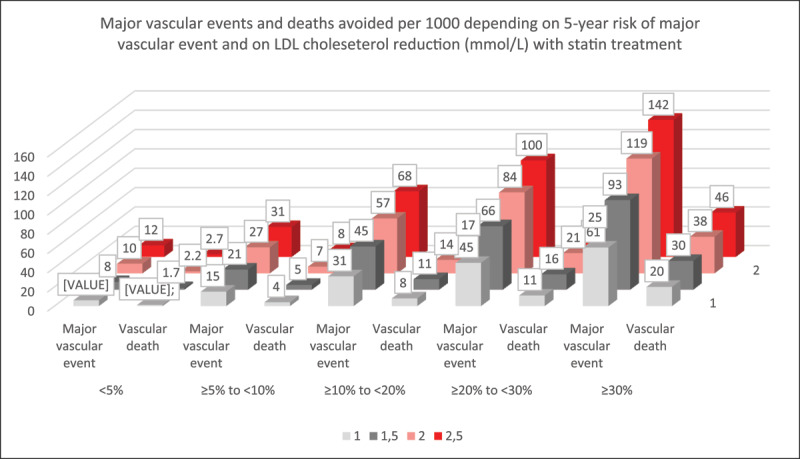
**Events avoided based on baseline absolute risk and absolute lowering of LDL-C (Duality of risk and LDL-C lowering as determinants of benefit from lipid lowering therapies).** Adapted from CTT Lancet 2012 The effects of lowering LDL cholesterol with statin therapy in people at low risk of vascular disease: Meta-analysis of individual data from 27 randomised trials [87].

LDL-C lowering has been shown to be beneficial in primary and secondary prevention with individuals at highest risk of atherothrombotic events such as myocardial infarction, stroke, revascularisation or cardiovascular death, deriving greater absolute risk reductions from LDL-C lowering (Figure 2). The importance of the timing of treatment initiation and the duration of that treatment in the disease course of atherosclerosis should now inform health policies and how they are implemented (as discussed later). Additionally, the vast majority of patients globally have insufficient LDL-C lowering to minimize their individual risk of ASCVD. In part this is due to the underuse of effective doses of more potent statins as first-line therapies, low use of combination therapies and poor adherence to lipid lowering regimens resulting in insufficient reductions in cumulative cholesterol exposure.

LDL-C lowering is beneficial not only in those with high cholesterol levels but rather in those most at risk of the major clinical manifestations of atherosclerosis with no lower limit of benefit observed from LDL-C lowering [10]. This is supported by genetic studies, prospective cohort studies, randomised trials of cardiovascular outcomes and imaging studies assessing atherosclerosis progression/regression. Recent evidence demonstrates that it is the magnitude of LDL-C lowering rather than how it is achieved that drives benefit [11]. Thus LDL-C lowering (and by proxy non-HDL-C and apo B) should be considered in all individuals at higher risk [12] identified as such by the presence of i) a prevalent/prior manifestation of ASCVD; ii) the presence of a high risk condition such as diabetes, chronic kidney disease, or extreme elevations of BP which increase ASCVD risk even in the absence of accompanying lipid abnormalities; iii) extreme elevations of LDL-C which have a genetic basis like heterozygous familial hypercholesterolaemia (FH); iv) or those with high-CV risk due to combined effects of multiple risk factors; v) isolated elevations in atherogenic lipoproteins including triglyceride rich lipoproteins (commonly referred to as atherogenic dyslipidaemia) or elevations in lipoprotein(a); vi) those with elevated burden of subclinical atherosclerosis [13]. This approach prevents recurrent events in those with prevalent ASCVD and incident events among those with high-risk conditions or high global risk.

## Measurement of blood lipids

Lipids can only be interpreted and become actionable as part of risk assessment or screening if they are measured in the first place. Reliance on total cholesterol misses the opportunity to identify other lipid abnormalities which may influence treatment decisions. Ideally, the full lipid profile total cholesterol (TC), HDL-C, and triglycerides (TG)—will be measured and LDL-C can be calculated using the Friedewald formula (or measured directly) and non-HDL-C can be calculated by subtraction (TC-HDL-C). Lp(a) should be included as part of the routine lipid profile. The accuracy of calculated LDL-C is unreliable when triglyceride levels are high >4.5 mmol/l (400 mg/dl) or when true LDL-C is low <1.8 mmol/l (70 mg/dl) (see online appendix). In these scenarios, analytical methods such as beta-quantification, which separates very low-density lipoprotein (VLDL) and intermediate density lipoprotein (IDL) allows accurate measurement of true LDL-C. However, this is expensive, and most laboratories do not have access to these methods, which remain largely the domain of research. Therefore, alternative approaches derived from large population cohorts and validated against beta-quantified measurements offer a pragmatic approach that can be implemented in laboratories around the world. Two new equations, the Martin-Hopkins equation and the Sampson equation have been developed, which allow estimation of LDL-C directly from non-fasting lipid profiles [14,15,16]. Both outperform Friedewald in the non-fasting setting with regard to triglyceride levels (see online Appendix). In direct comparisons, the Martin-Hopkins equation is the most accurate method of the three equations with directly measure LDL-C as the standard [17]. Both new equations can be integrated into regular use by pathology laboratories for reporting LDL-C values in LMICs (eTable 1).

Conventionally, lipids have been measured in blood samples obtained after eight hours of fasting, to minimise the effects of postprandial changes in TG which influence LDL-C calculations. However, using one of the newer formulas to estimate LDL-C can be achieved in non-fasting samples. Alternatively, total atherogenic cholesterol content (non-HDL-C) or total number of atherogenic particles (apo B) can be obtained from non-fasting samples offering a more comprehensive measure of atherogenic risk, especially when apo B and non-HDL-C are discordant with LDL-C such as in conditions associated with insulin resistance, such as diabetes, obesity and high TG. Where appropriate laboratory facilities exist, apo B should in time become the default measure of total atherogenic lipid burden. Pragmatically, we have multiple approaches to measuring atherogenic lipid risk (LDL-C, non-HDL-C or apo B), and any of these are better than none at all. Therefore, in resource poor environments the focus should be on making some form of cholesterol testing available as a starting point for eventually achieving best practice (eTable 2).

Finally, there is consistent data irrespective of ethnicity that higher levels of lipoprotein(a) are likely causally associated with a higher risk of ASCVD (typically > 125 nmol/L or 50 mg/dl which occurs commonly). As lipoprotein(a) cannot be estimated from other lipid measures it has to be measured directly, preferably using an isoform independent assay reporting values in terms of concentration (e.g., nmol/L) rather than mass (e.g., mg/dL). As levels do not change much over a lifetime, except in women where values further increase after menopause, or in acute inflammatory states, a single measurement once in a lifetime in adults is useful to reliably exclude significant elevations [18].

## Overall approaches to prevention

### Primordial prevention and population health

The first step in the public health approach to ASCVD prevention is to change the milieu that promotes risk factor development as early as possible [19]. While primary prevention is about treating risk factors to prevent ASCVD, primordial prevention is avoiding the development of risk factors in the first place. It begins with changes in lifestyle, environmental and social conditions to prevent risk factor development from birth. Many risk factors have their origins early in life since this is the time when lifestyles are formed. Whilst genetics may be considered the ‘loaded gun’, environment and lifestyle effectively ‘pull the trigger.’ Many of these changes require policy changes, affordable healthy life choices and improvements in health literacy. Population level changes at a time of maximal developmental plasticity offer the best hope of setting individuals on a more favourable trajectory long-term towards better cardiovascular health. Recommendations regarding this remain the same as in the 2017 Roadmap; public health interventions should incentivise a healthy lifestyle. These can simply be put as keep physically active, an important necessity as many jobs have a sedentary nature, avoid tobacco use, maintain ideal body weight for ethnicity, reduce salt consumption and where feasible adopt the traditional Mediterranean-type diet with substitution of monounsaturated and polyunsaturated for saturated fat [20,21]. In particular, plant-based foods should be encouraged and incentivised [22,23,24]. Specifically, these approaches should focus on children or young adults, that is, start as early as possible when it comes to primordial prevention.

### Primary Prevention

#### Current approach: 10-year ASCVD risk assessment

In the absence of high-risk conditions such as genetic dyslipidaemias (which include familial hypercholesterolaemia), diabetes mellitus, chronic kidney disease, a global approach to assess/predict 10-year risk of fatal and non-fatal ASCVD events should be used as those at highest risk derive greater absolute risk reductions from lipid lowering therapies (with smaller numbers needed to treat). These approaches should be region specific where available data allow, re-calibrated and updated as appropriate incorporating additional relevant risk factors that may emerge and allowing for secular trends to provide more reliable estimations of absolute risk. Risk calculators which use fatal events only disenfranchise younger individuals and women and their use is discouraged in favour of calculators assessing both fatal and non-fatal events. The decision of who to screen for ASCVD risk, and which risk threshold to use to initiate pharmacological treatments if healthy diet and lifestyle changes do not sufficiently attenuate risk is a matter for national (or local) policy which considers available resources. In the presence of conditions such as previous manifestations of ASCVD, genetic dyslipidaemias which include familial hypercholesterolaemia, diabetes mellitus and chronic kidney disease, risk calculators are not necessary to initiate therapy [25,26]. Categorising individuals into different risk categories provides the rationale and helps tailor the intensification of any intervention.

Global ASCVD risk calculators could be in the form of charts or preferably available as a web-based tool or Apps on a smartphone to be easily accessible. These should not only be used to provide estimates of risk (whom to potentially treat) but could be used to help shared decision-making, and where feasible, provide visualisation not only of potential risk but also of the magnitude of any potential benefit obtained from reductions in atherogenic lipids (and other factors). Ideally these could be automatically integrated into electronic medical record systems where the required data elements are available. This could aid/improve patient understanding and acceptance plus adherence to a given treatment regimen. Examples of contemporary calculators are shown in eTable 3. All risk prediction tools have strengths and limitations and should be used in conjunction with clinician judgement. As shown in the recent SCORE2 risk calculator [25], baseline risk in part related to societal, environmental and potentially genetic differences varies widely across geographical regions. Hence risk calculators which have been closely calibrated to the population of interest should be used where feasible, to avoid over or underestimation of risk. This has been done for different regions with the new WHO risk calculator [27]. This inevitably means that for the same level of risk factors, predicted event rates will vary, hence the threshold for treatment may vary to avoid treating too many or too few people. That said, in many countries the rate of CVD in indigenous or first world communities is very high and risk scores don’t work so well – so the implications for lipid lowering are more important.

In general, formal 10-year risk assessment should not be used among individuals with high-risk conditions, such as FH (as discussed later), type 2 diabetes or chronic kidney disease. However, risk of ASCVD is a continuum even among individuals with high-risk conditions such as diabetes. It is now recognised that diabetes is not a ‘coronary heart disease risk equivalent’, but rather absolute risk varies considerably by presence or absence of microvascular disease/end organ-damage, duration of diabetes and presence or absence of additional risk factors, duration or high CAC score [28]. Diabetes is included as a variable in some risk equations but can also be pragmatically categorised in those with moderate, high or very-high risk, that is, the presence of diabetes alone places an individual as a minimum into the moderate risk category [26,29]. Currently, the severity of renal impairment may be used to categorise patients into high (eGFR 30-60) or very high-risk categories (eGFR < 30).

From a pragmatic perspective for day-to-day practice, primary prevention includes those without overt major clinical manifestations of ASCVD. Imaging, especially in the coronary arteries like coronary artery calcium scores or computed tomography coronary angiography, is being used increasingly during the course of routine clinical practice, although cost has limited their use in LMICs. Where evidence of atherosclerosis is found, most guidelines recommend using this as a risk enhancer in the setting of primary prevention, thus evidence of risk enhancing factors should result in more aggressive control of risk factors due to reclassifying these people into a higher risk category.

### Future approach: Lifetime risk estimation

The main implication of the cumulative exposure hypothesis is that maintaining low levels of LDL-C throughout life reduces cumulative exposure to the number of lipoprotein particles that become trapped within the artery wall over time, thus slowing the rate at which atherosclerosis develops and delaying the age at which the underlying plaque burden is large enough to increase the risk of having an acute ASCVD event. However, to maximize the benefit of reducing plaque burden by reducing cumulative exposure to LDL-C, it is also necessary to reduce exposure to other causes of arterial wall injury that increase the risk of having an acute event at all levels of plaque burden. Therefore, the cumulative exposure hypothesis suggests that the most effective way to reduce the lifetime risk of ASCVD events is by reducing cumulative exposure to LDL-C to slow atherosclerotic plaque progression, while also reducing cumulative exposure to other causes of arterial wall injury including elevated blood pressure, diabetes, and tobacco smoking.

Very long-term prospective follow-up of the Framingham Heart Study demonstrates that persons who maintain low levels of LDL-C and blood pressure throughout their lives have a very low lifetime risk of atherosclerotic cardiovascular events [30]. The PDAY risk score based on the relationship of risk factors to measured atherosclerosis in 15–34 year-olds strongly predicts premature ASCVD [31]. In addition, Mendelian randomization studies demonstrate that persons who are naturally randomized to genetic variants associated with lower lifetime exposure to LDL-C have a substantially lower lifetime risk of ASCVD [32,33]. The same observation is true for BP. The latter studies agree closely with the 80% reduction in cardiovascular morbidity and mortality observed in Finland over the past 40 years [34]. Beginning in the 1970s, Finland initiated a coordinated public health programme to reduce cardiovascular morbidity and mortality by reducing exposure to saturated fats to lower plasma cholesterol levels, and by discouraging the practice of preserving meat with cured salts to lower blood pressure levels [34]. Over the following 40 years, this programme resulted in a reduction of 1.5 mmol/L (58 mg/dL) in the population mean level of total cholesterol, an 8.7 mmHg reduction in the population mean level of systolic blood pressure (SBP), and a corresponding 80% reduction in cardiovascular morbidity and mortality. Assuming that most of the total cholesterol reduction was due to reduction in LDL-C in response to lower consumption of saturated fats, the magnitude of this reduction in cardiovascular morbidity and mortality is nearly identical to what would have been predicted by long-term exposure to the same magnitude of lower LDL-C and SBP in Mendelian randomization studies, thus providing powerful real-world evidence to support the cumulative exposure hypothesis.

Although some guidelines have referenced the value of lifetime risk [25,35,36], current clinical practice does not focus on lifetime risk [37], or on reducing cumulative exposure to the causes of ASCVD. Instead, current clinical practice guidelines recommend both informing the decision to initiate LDL-C lowering therapies, and titrating the intensity of those therapies, based on a person’s estimated short-term risk of developing an acute cardiovascular event over the next 10 years (29). However, short-term risk estimating algorithms are mathematically dominated by age because acute atherosclerotic cardiovascular events tend to occur later in life after a person develops a substantial underlying atherosclerotic plaque burden. As a result, informing the decision to initiate LDL-C lowering therapy based on short-term 10-year risk has the practical effect of inviting us to wait until a person develops a large atherosclerotic burden before initiating therapy to lower LDL-C to slow the progression of atherosclerosis.

**Figure 3:**
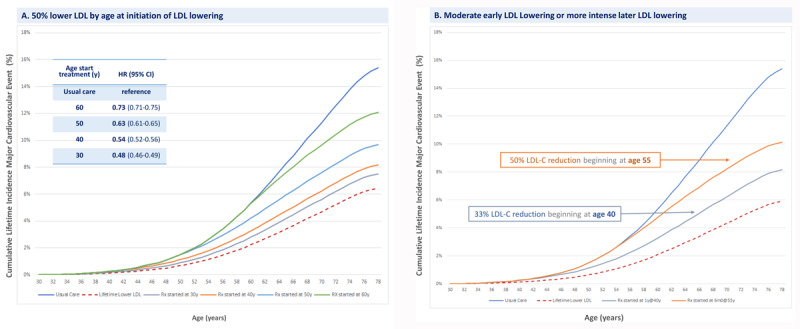
**Benefit of reducing cumulative exposure to LDL on the lifetime risk of atherosclerotic cardiovascular disease.** Panel A shows the effect of reducing LDL by 50% from a population median of 3.5 mmol/L (135 mg/dl) resulting in an absolute difference of 1.75 mmol/L (67.7 mg/dL) on the lifetime risk of experiencing a major atherosclerotic cardiovascular event (defined as fatal or non-fatal myocardial infarction, fatal or non-fatal ischemic stroke, or coronary revascularization) if LDL lowering is started at ages 30, 40, 50 or 60 years and continued up to age 80 years as compared to either no LDL reduction of lifelong exposure to the same magnitude of lower LDL. Panel B shows the effect on the lifetime risk of experiencing a major atherosclerotic cardiovascular event up to age 80 years from reducing LDL by 33% beginning at age 40 years, or by 50% beginning at age 55 years. Greater benefits are observed if LDL-C lowering is begun at an earlier age.

Indeed, current clinical practice guidelines explicitly focus on using pharmacologic therapy to lower LDL-C to prevent atherosclerotic cardiovascular events among persons with a high estimated 10-year risk of experiencing an acute ASCVD event; and on intensification of pharmacologic LDL-C lowering therapies to achieve even lower LDL goals among persons who have already experienced an ASCVD event. Achieving these more intense plasma LDL goals almost always require the use of two or more pharmacologic LDL-C lowering therapies. Recent advancements in embedding causal inference and causal effects into artificial intelligence and machine learning algorithms (‘causal AI’) permit an estimation of the effect of lower LDL-C or systolic blood pressure (SBP) in discrete time-units of exposure, conditional on previous cumulative exposure to account for the amount of plaque burden and arterial wall injury that has accumulated prior to the initiation of interventions to lower LDL-C or SBP. This method allows an estimate of the benefit of lowering LDL-C, SBP or both beginning at any age (and extending for any duration of time) on the risk of developing an acute atherosclerotic cardiovascular event. Studies using these causal AI methods suggest that there is a stepwise increase in the reduction in the risk of atherosclerotic cardiovascular events for each decade earlier that LDL-C lowering is initiated [32] (Figure 3).

These studies suggest that modest sustained reductions in LDL-C beginning earlier in life are associated with a lower risk of ASCVD events at all ages as compared to more intense LDL-C lowering started later in life. Indeed, these studies suggest that ‘residual risk’ of experiencing an acute ASCVD event despite intense LDL-C lowering initiated later in life may be explained by the extent of the plaque burden that accumulates prior to the initiation of LDL-C lowering therapy. Although aggressive LDL-C lowering initiated later in life can slow (or perhaps even arrest) plaque progression, the plaque burden that accumulated prior to the initiation of LDL-C lowering still persists and may disrupt the subsequent formation of a thrombus overlying the disrupted plaque, obstructing blood flow and leading to an acute cardiovascular event. As a result, reducing the cumulative exposure to LDL-C as a strategy to slow the progression of atherosclerotic plaque may both substantially reduce the lifetime risk of ASCVD and substantially reduce the residual risk of acute atherosclerotic events if implemented globally.

### Familial hypercholesterolemia

Familial hypercholesterolaemia is an autosomal co-dominant condition which results in life-long elevations in LDL-C from birth leading to premature cardiovascular disease (from age 30 in men and age 40 in women) and premature deaths. Heterozygous FH (HeFH), results when an individual inherits one affected gene from a parent. Several recent studies have emerged that should inform future public health strategies directed towards early case finding and initiating treatments early in the life course.

The global prevalence of HeFH is 1:311 and is present in all WHO regions at similar levels [38]. However, the frequency in communities with founder effects is higher, being as high as 1:70 [39]. Even though it affects every WHO region, there is little data from as many as 130 countries, meaning that HeFH may not even be recognised and is thus a missed opportunity. The FHSC (Familial Hypercholesterolemia Studies Collaboration) global registry demonstrated that HeFH diagnosis globally occurs in the fourth decade of life with only 2.1% diagnosed before the age of 18 years, with age of diagnosis in the Asia-Pacific region worst, underscoring the need for system-wide changes enabling early diagnosis [40]. At diagnosis, 17.4% had coronary artery disease (CAD), 2.1% stroke and 5.2% peripheral arterial disease with 11.3% having premature CAD [40] consistent with data suggesting that among patients with premature CAD the prevalence of FH could be as high as 1:17–25 [38].

The lack of widespread availability of genetic testing means that the commonest method of diagnosis globally is using clinical criteria with ~75% using the Dutch Lipid Clinic Network criteria (DLCN) [40], consisting of biochemical measurements, physical examination and family history. Women are diagnosed several years later than men and are less likely to have CVD at diagnosis. This means that contributions (from premature ASCVD) to the DLCN score will be absent, making LDL-C, physical examination and family history even more important, to even consider a diagnosis of FH in women. Furthermore, with increasing age, metabolic causes of elevated LDL-C become more common. As the prevalence of risk factors such as hypertension, diabetes and increased BMI increase with age (40), if patients with FH are identified earlier there is a greater chance that unhealthy lifestyles and other behaviours might be avoided reducing the likelihood of lifestyle associated risk factors later in life. Treatment beginning in adolescence dramatically reduces premature myocardial infarction and death in this condition [41]. Case finding through screening in childhood (as discussed later) is attractive for multiple reasons, not least because LDL-C is less likely to be influenced by metabolic disorders in this age group, increasing the likelihood that elevated cholesterol has a genetic basis.

Though cholesterol lowering treatment is effective, most adult patients with HeFH will likely require at least two therapies. The FHSC registry demonstrates that less than 3% of patients achieved LDL-C levels of < 1.8 mmol/L (70 mg/dL). At present, though statins are generally used, ezetimibe is only used in about 24.6% and PCSK9 monoclonal antibodies (MAbs) in 3%. As patients with HeFH usually have an LDL receptor (LDL-R) mutation, the normal gene can be upregulated with statins and LDL-R lifecycle enhanced with therapies that lower free PCSK9. In this regard inclisiran with twice yearly dosing may be convenient offering similar efficacy to MAbs which require dosing every two weeks, when added to statins in genetically confirmed HeFH [42].

Homozygous FH (HoFH) occurs in approximately 1:300 000. Affected individuals have extreme elevations in LDL-C – typically > 13 mmol/L (500 mg/dL) – and manifestations of ASCVD or aortic or supra-aortic stenosis before the age of 20 which is invariably fatal by aged 40 if untreated [43]. Those affected carry two affected genes in pathways related to cholesterol metabolism. Recently available data on HoFH from 38 countries showed that the average age of diagnosis was 12 years, with average LDL-C levels of 14.7 mmol/L (570 mg/dL) and with 9% already having evidence of ASCVD or aortic valve disease at diagnosis [44]. There are significant differences in how patients are treated between high- and low-to-middle-income countries which impacts health outcomes. Patients in high-income countries (HICs) had LDL-C levels on treatment of ~4 mmol/L (155 mg/dL) and were more likely to receive newer therapies with four or more drug combinations than in non-high-income countries. The latter by contrast mostly use two drug combinations and had on treatment LDL-C levels of about 9 mmol/L (350 mg/dL). The average age of first cardiovascular event in low-middle income countries was at 24.5 years and at 37 years in high income countries. Cardiovascular risk related to high-LDL-C in part could be attenuated with an earlier age of diagnosis as well as using four or more lipid lowering treatments, including apheresis. Notably, as many of these patients have severely diminished LDL-R function, hence traditional therapies that work through the LDL-R pathway like statins, ezetimibe and PCSK9 inhibitors may not achieve the magnitude of LDL-C lowering necessary to reduce atherosclerosis progression. This necessitates the need for newer therapies that are independent of the LDL-R like lomitapide and evinacumab. These therapies are currently extremely expensive but potentially lifesaving.

### Secondary Prevention

As the risk of recurrent ASCVD events and progression of atherosclerosis is highest in individuals with pre-existing or prevalent manifestations of ASCVD, pharmacological interventions for cholesterol lowering need to be initiated without formal risk estimation. This means statins usually of high potency (with the aim to achieve at least 50% reduction in LDL-C) are used irrespective of untreated cholesterol levels rather than starting at low doses and titrating up. This is particularly important among those who experience an acute atherothrombotic event and are at greatest risk of further events [45]. Just as with primary prevention, among ASCVD patients, risk varies considerably [46,47]. As more recent global guidelines have recommended lower cholesterol goals for these patients, it is now clear that this inevitably means that statin-based monotherapy will not help the majority of patients reach lower LDL-C levels. For instance, the new ESC/EAS 2019 goals of <1.4 mmol/L (55 mg/dl) are likely to be achieved by only ~1/5 individuals and with only ~1/2 achieving goals of <1.8 mmol/L (70 mg/dl) [48]. Globally there is an underutilisation of combinations of lipid lowering treatments [48] which are inevitably necessary in order to achieve the new recommended cholesterol levels. Although newer add on therapies are available, their cost and uncertainties in everyday practice as to who benefits most when healthcare resources are scarce have impacted their widespread use. In this regard, estimation of 10-year risk with the SMART risk equation is globally the most validated tool for assessing absolute risk and thus allows estimation of absolute benefit [46,49]. A schematic shows how among 13 patients with ASCVD, risk varies based on demographics and co-morbidities, Absolute benefits of anti-coagulants depends upon the starting global risk. The absolute benefits of further LDL-C lowering through PCSK9 lowering therapies depends upon the global risk as well as on the treatment levels of LDL-C, for instance on statins and how much further it is lowered by adding on non-statin lipid lowering therapies eTable 4. The SMART tool provides up to 10-year quantitative assessments of risk and potential benefit and is available as an online tool. Where this is not available, simple qualitative tools like the TIMI Risk Score for Secondary Prevention (TRS2P) in those with established ASCVD, have been shown in several studies to differentiate by a factor of three between those at very high risk from those at much lower risk and who derive greater absolute benefits from further cholesterol lowering [26,50].

## Lipid modification therapy to prevent ASCVD

As the risk of incident ASCVD or recurrent ASCVD events is multi-factorial, a global approach is required to prevent adverse clinical outcomes. As such, modification of all risk factors starting with lifestyle are needed, and for those higher risk primary prevention individuals (to prevent incident disease) or those with prevalent clinical ASCVD, using pharmacological interventions to target different causal pathways offers independent and additive benefits [51,52]. The greater the baseline absolute risk the greater the absolute benefit from any intervention, hence the aim of lipid modification is first to lower all atherogenic lipids (LDL-C, non-HDL-C and apo B), and second to match the degree of lowering and achieved on treatment LDL-C levels to the level of risk, that is, those at greatest risk should achieve greater reductions in and have lower on treatment levels (eTable 5).

### Pharmacological lipid lowering therapies

#### LDL-C

Healthy diet and lifestyle have been covered in prior roadmaps. For some these are insufficient. Details of currently available and future therapies, their mechanisms of action and where they should be used are available in detail in the supplementary material although they may not all be available in all regions of the world. Therapies fall into two categories; oral agents which require daily dosing and injectable therapies which require less frequent dosing. Statins remain first line therapy. They are generic and generally safe and available throughout the world. They should be initiated at the outset using effective doses that offer the greatest reductions in LDL-C to avoid delays in treatment escalation (for instance 40–80 mg atorvastatin, 20–40 mg rosuvastatin, i.e., regimens that could provide ~50% lowering). Routine monitoring for adverse effects, such as liver function, are not required unless clinically indicated. Measuring LDL-C is needed to assess response to therapy and adherence. Multiple studies have shown a lack of association between achieved LDL-C levels and adverse events. The commonest adverse effect (myalgia) may be mitigated by cessation of therapy and re-challenge at a similar dose or by a reduction in dose of the statin or reduction in the frequency of dosing (once weekly, twice weekly, alternate doses) or switching to an alternative statin [53,54,55]. Ezetimibe is also now available as a generic therapy in many parts of the world and in many countries as a combination pill with statins. Bempedoic acid is the third oral agent which has now become available in north America, Europe and elsewhere and the combination of this with ezetimibe offers similar reductions in LDL-C to a high-intensity statin alone [56], which is a possible option in those with intolerance to statins. Injectable therapies against PCSK9 include monoclonals (mAbs), which require twice monthly or monthly dosing, and inclisiran, a small interfering RNA (siRNA) therapeutic which requires twice yearly dosing. These are now available in many countries. Uptake of injectable therapies to date has largely been limited by cost, as these are more expensive than oral therapies.

#### Combination therapies

An inevitable consequence of the lowering of recommended cholesterol goals around the world is the need for the use of multi-drug combinations targeting different pathways in cholesterol regulation. We have increasingly recognised that many people with hypertension require multiple agents to achieve effective lowering. We need to acknowledge this may be the same for cholesterol and ask the question of how we can develop smart ways to do this? The current stepwise approach often results in clinical inertia, which in turn results in inadequate control of cholesterol, demonstrated almost serially by recurrent surveys and registries [57]. Oral combinations of two therapies are already available in the form of a single pill; statins (at various doses) and ezetimibe and bempedoic acid plus ezetimibe which may reduce pill burden and help long-term adherence. Potential combinations of oral and injectable therapies and the potential LDL-C reductions achievable are shown in Table 1.

**Table 1:** Potential LDL-C reductions achievable through different combinations of lipid lowering treatments.


	EXPECTED LDL CHOLESTEROL REDUCTION			

PATIENT TYPE	STATIN TOLERANT		STATIN INTOLERANCE	

**Achieveable Reductions**	**≥60%**	**≥80%**	**≥35%**	**≥60%**

**Potential Combinations**	Rosuvastatin 20–40 + Ezetimibe 10	Rosuvastatin 20–40 + Alirocumab/Evolocumab (+ Ezetimibe 10)	Ezetimibe 10 + Bile acid abs	

	Atorvastatin 40–80 + Ezetimibe 10	Atorvastatin 40–80 + Alirocumab/Evolocumab (+ Ezetimibe 10)		Ezetimibe 10 + Alirocumab/Evolocumab

	Rosuvastatin 5–10 + Alirocumab/Evolocumab			

	Atorvastatin 10–20 + Alirocumab/Evolocumab			

	Rosuvastatin 5–10 + Inclisiran	Atorvastatin 40–80 + Inclisiran (+ Ezetimibe 10)	Bempedoic acid 180 + Ezetimibe 10	

	Atorvastatin 10–20 + Inclisiran	Rosuvastatin 20–40 + Inclisiran (+ Ezetimibe 10)		Bempedoic acid 180 + Ezetimibe 10 + Evolocumab, Alirocumab or inclisiran

	Atorvastatin 20 + Ezetimibe 10 + Bempedoic acid 180			Ezetimibe 10 + Inclisiran


Reproduced from Ray KK *Eur Heart J*, ehab718 [76]. DOI: https://doi.org/10.1093/eurheartj/ehab718

#### HeFH

Adults with heterozygous FH usually have untreated LDL-C values > 4.9 mmol/L (190 mg/dL) and therefore will need potent cholesterol lowering therapies to reduce LDL-C at least 50% and to attain recommended goals [29]. Observational studies have shown that reductions in LDL-C between 30–50% prevent the risk of ASCVD in people with FH [41,58]. Therapy should be started with a high potency statin (40–80 mg atorvastatin or 20–40 mg rosuvastatin) usually in association with cholesterol absorption inhibitors like ezetimibe. The availability of generic, low-cost combinations of ezetimibe with potent statins should facilitate LDL-C lowering in these individuals. Bempedoic acid may also add LDL-C lowering on top of the latter. Despite this, most patients will not attain recommended LDL-C goals due to their elevated baseline values [59]. PCSK9 directed therapies, either mAbs or siRNA should be used to further lower LDL-C in those at higher risk (previous ASCVD, elevated subclinical coronary atherosclerosis plaque burden, those with multiple risk factors like smoking, diabetes, hypertension, low HDL-C, high lipoprotein(a) among others) or who persist with LDL-C levels above recommended goals [29,43].

#### HoFH

Although statins and ezetimibe underpin therapeutic approaches to LDL-C management in HoFH, they will be insufficient for most necessitating the need for approaches that are independent of the function of the LDL-R. Lomitapide is a small molecule requiring daily dosing which targets the microsomal triglyceride transfer protein thus inhibiting very low density lipoprotein (VLDL) assembly, resulting in 38–50% lowering of circulating LDL-C levels [60]. Injectable therapies available include PCSK9 directed mAbs which could reduce LDL-C by ~25–45% [61,62], but have no effect if LDL-R function is severely reduced. Evinacumab is a monoclonal antibody against angiopoietin-like peptide 3 (ANGPTL3), which can reduce LDL-C by ~49% irrespective of genotype [63]. Reductions in LDL-C with evinacumab were observed irrespective of background lipid-lowering therapies (mAbs against PCSK9, lomitapide and lipoprotein apheresis). However, this treatment requires monthly intravenous infusion and observation over approximately two hours and therefore requires appropriate infrastructure which may limit its more generalised use. Lipoprotein apheresis is highly effective at reducing LDL-C but is invasive and requires specialised centres with appropriate capability to administer this service which may limit its wider use.

### Triglycerides

Triglyceride (TG) elevations often reflect the spectrum of lifestyle related metabolic dyslipidaemia. Hence, weight loss, diabetes control, alcohol reduction and carbohydrate reduction are important means to lower TG levels. If TG levels fall, the cholesterol within TG rich lipoproteins will also fall. Most guidelines recommend more intensive non-HDL-C or apo B lowering for patients with high triglycerides for reasons discussed earlier, due to an absence of clear benefit from therapies such as fibrates in individual contemporary trials although meta analyses suggest some benefits in the high TG/low HDl-C phenotype [12]. However, among patients with high TG the use of icosapent ethyl resulted in significant reductions in CV events despite only modest TG lowering suggesting that any clinical benefit may be through non-lipid related pathways [64]. Some guidelines now recommend high dose icosapent ethylicosapentethyl as a potential option to reduce CV risk among high-risk patients (ASCVD or diabetes and multiple risk factors) with TG (1.5–5.6 mmol/L or 135–499 mg/dl) and on statin therapy [26,29].

### Lipoprotein(a) and Lp(a)

Lipoprotein (a) should be measured at least once in a person’s lifetime. Management of individuals with high Lp(a) currently focuses on control of other major risk factors to reduce ASCVD risk [65]. Lipoprotein apheresis is often used to reduce severe elevations in Lp(a) and more modest reductions of about 20–30% are achievable through PCSK9 directed therapies [66]. To what extent Lp(a) lowering with these therapies confers additional benefit beyond the LDL-C lowering is uncertain although post-hoc analyses using post randomisation changes suggest there may be benefit [67] from PCSK9 mAbs from Lp(a) lowering independent of the LDL-C lowering effects. Trials focused on specifically lowering Lp(a) by 80% or more with RNA based therapies are ongoing to evaluate this.

## Implementation of the 2017 WHF Cholesterol roadmap

Since the original WHF Cholesterol Roadmap was published, it has been adopted in several countries around the world. In particular, national roundtables have been instrumental in adapting the WHF Roadmap to fit local conditions.

For example, in Colombia, the Colombian Society of Cardiology and Cardiovascular Surgery organized a roundtable in November 2017, in collaboration with the WHF, the Ministry of Health, academia and Civil Society. This meeting resulted in the adoption of the ‘Colombian Manifesto Against Heart Attack’, with the ambition to achieve 7000 fewer heart attacks per year in the country by 2025. To achieve this goal, three priority actions were decided for cross-institution implementation: Creation of the National Registry of Infarctions in Colombia – Programa Bandera Roja, restructuring of cardiac rehabilitation in all of healthcare institutions programmes and priority implementation of cholesterol clinical guidelines for the management of high-risk patients in all service provider institutions in the country. The main actors of the health system committed to encourage a public policy that leads to the reduction of deaths from cholesterol in Colombia, to not only decrease mortality but also improve the quality of life of patients and the sustainability of the health system.

In Saudi Arabia, the Saudi Heart Association, in collaboration with the WHF and the Saudi Health Council, convened a multi-stakeholder group of senior officials from both government and non-government agencies at a roundtable in Riyadh in December 2017. The national roundtable identified a series of country-specific roadblocks to better CVD management at patient-, clinician- and health care system- levels. Following this cholesterol roundtable, the Saudi Heart Association planned to conduct a Delphi survey using the Roadblock document to identify top country-specific priority areas and feed the results of the survey into a follow-up roundtable focusing on solutions to overcome the main barriers. The next roundtable will include interactive brainstorming workshops and will result in an outcome document highlighting solutions and, reflecting the input of key stakeholders for better cholesterol management in Saudi Arabia. The report will be submitted to the Saudi Health Council.

In 2018, in the Philippines, the WHF and the Philippine Heart Association convened a multi-stakeholder group of 56 experts and speakers in cardiology, neurology and family medicine to identify country-specific roadblocks to and solutions for better management of cardiovascular disease (CVD) and cholesterol at patient-, physician- and health care system- levels. Based on the outcomes of presentations and panel-discussions, a plan of action on cholesterol for the Philippines was developed, with a focus on concrete steps that can be taken in-country to overcome these roadblocks. Priority solutions identified included strengthening collaborations for patient screening, reinforcing continuing medical education activities, improving access to health facilities among poor or remote populations and updating national dyslipidaemia guidelines. In addition, the Philippine Heart Association agreed to continue its monthly/bi-monthly screening programmes nationwide, depending on funding availability.

In Belgium, the Belgian Heart League and the WHF convened a National Stakeholder Roundtable in December 2021 to agree on needs and priorities in terms of prevention and detection of hypercholesterolemia. Concrete and feasible actions are being coordinated by the Belgian Heart League as of 2022. Agreed action points include to work closely with FH Europe in order to join forces with other EU countries and implement FH screening nationwide.

## Current roadblocks and potential solutions

### Who was surveyed?

**Figure 4:**
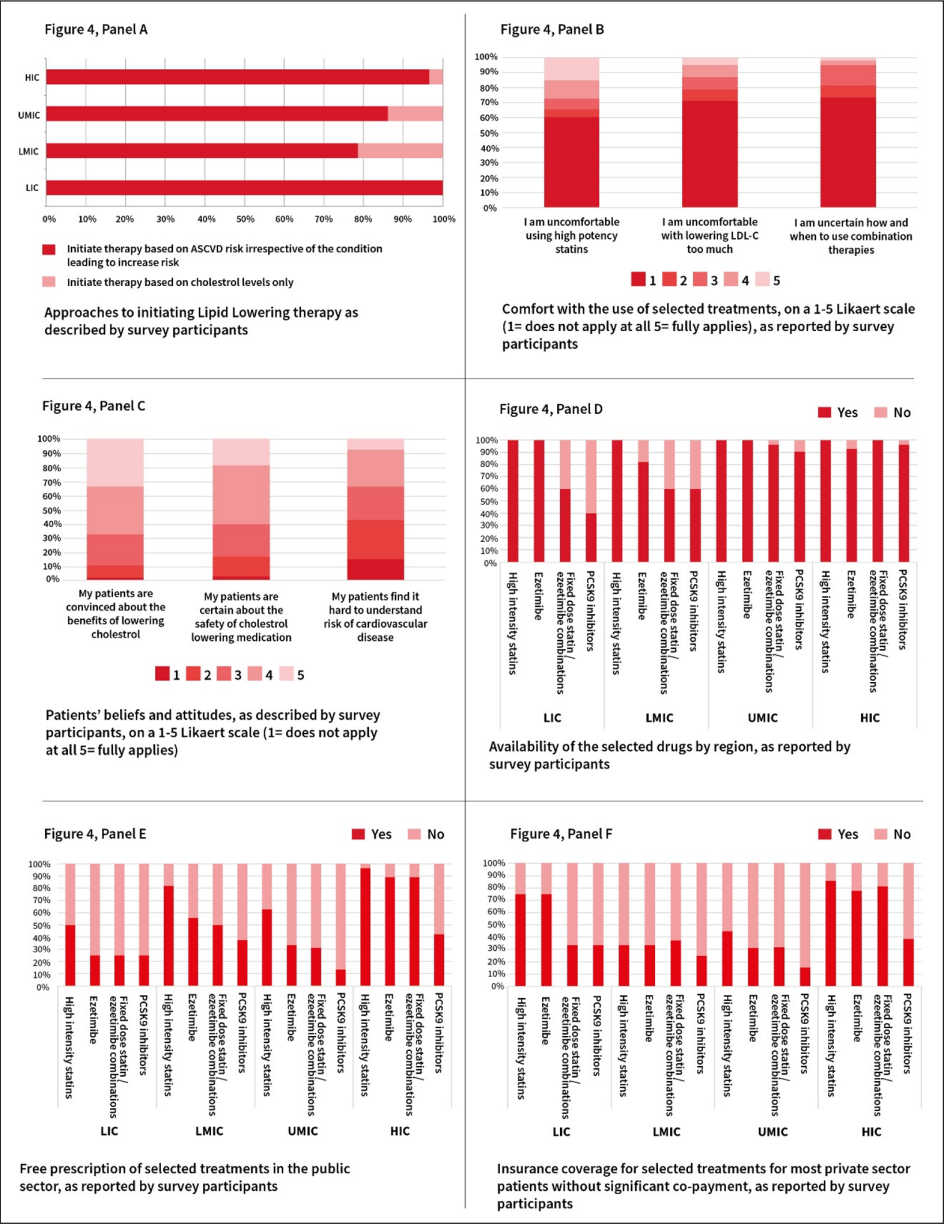
**Selected WHF member survey responses (based on responses from 38 countries from all WHO regions)** *© World Heart Federation*.

In July and August 2021, the WHF conducted an online consultation of all WHF members using snowball sampling to include regional members as well as national representatives (Figure 4, Panels A–F). This online survey included questions on clinical practice as well as questions on the roadblocks and solutions which had been identified in the original WHF Cholesterol Roadmap. In total, responses were collected from 38 countries. The highest number of responses were collected in the WHO Region for the Americas, followed by the European Region. Respondents predominantly worked in an urban setting with 40% working in public hospitals, 23% in private hospitals and 18% in outpatient clinics only. Other work settings included government organisations, research organisations and NGOs. Seventy percent of the respondents were cardiologists, followed by lipid specialists, endocrinologists, nurses, nutritionists, researchers, and health advocates. The specialities represented in part reflect WHF membership, but perhaps also point to major barrier in ASCVD prevention, namely healthcare systems focused on treating disease based in secondary and tertiary care rather than in primary care with a focus on preserving health. Furthermore, those surveyed may represent the best-case scenario of practitioners who are engaged and up to date with cholesterol management and ASCVD prevention.

### Which guidelines are used by WHO region

Overall, most respondents recognize that they follow an ASCVD prevention guideline to manage LDL-C. Broadly by WHO region, the European and African WHO regions relied mostly on the ESC/EAS guidelines, the Eastern Mediterranean and the Western Pacific WHO Regions on the ACC/AHA guidelines, and the picture was more contrasted in the WHO Region for the Americas and the Southeast Asia Region (details in Supplementary Appendix). Overall, across WHO income regions the ESC Score charts were the most commonly used. The WHO has published CVD risk prediction charts for each WHO region. Hence prior roadblocks such as the absence of risk tools for regions not studied in ESC or ACC/AHA risk calculators such as Africa or Latin America should not be obstacle. Prior uncertainty about the accuracy of risk prediction tools potentially which may have resulted in no risk prediction tool being used whatsoever, or when used over/underestimation of risk can now be overcome.

### Perceptions about lipid lowering therapies and cholesterol

Clinician and patient perceptions about the need or effectiveness of therapies could be an impediment to better management of cholesterol and ASCVD risk. An important finding is that 93.9% of the respondents reported initiating lipid lowering therapy based on ASCVD risk irrespective of the conditions leading to increased ASCVD risk. Only 12.2% reported initiating therapy based on cholesterol levels alone. This trend was seen in all WHO income regions (Figure 4, Panel A). Overall, around 27% of respondents are uncomfortable with prescribing high potency statins. Notably, 60% of the respondents from LMICs reported being uncomfortable prescribing high potency statins. Conversely, in HICs 65% reported being fully comfortable with the use of high potency statins (Figure 4, Panel B). This represents a significant barrier to tackling ASCVD globally as the majority of the global population reside in LMIC. Across income regions, approximately 25% of the respondents felt somewhat or highly uncomfortable with lowering LDL-C ‘too much’. A minority of respondents felt uncertain about how and when to use combination therapies. Given the low use of combination therapies globally, this suggests that there is a wide gap between self-reported awareness of what to use and when to use combination therapy and correct application.

Patient perceptions collated from respondents suggest that two-thirds of the respondents felt that their patients were convinced about the benefits of lowering cholesterol and around 20% felt that patients were not convinced about these benefits. Similarly, around 60% felt that patients were convinced about the safety of cholesterol lowering medications. However, among LMICs, 70% of the respondents were unsure or felt this was not the case. Finally, 80% of the respondents in low-income countries (LICs) felt that patients find it hard to understand the risk of ASCVD (28% in HICs) (Figure 4, Panel C). Taken together much more work is needed to improve health literacy including a greater need for patient/public engagement as well as shared decision making. Limitations of our survey merit consideration. In particular, the sampling may not be fully representative as some surveyed did not respond and the ‘perception’ of physicians about patients were considered instead of patients being asked directly.

### Availability and access to different lipid lowering therapies

High-intensity statins and ezetimibe were reported to be available by most responders independent of WHO income region. In LICs, 40% of the respondents pointed to the lack of availability of fixed dose statin/ezetimibe combinations, and 60% reported that PCSK9 inhibitors were not available. In LMICs, the lack of availability of ezetimibe was reported by 20% of the respondents, and the corresponding figure for fixed dose statin/ezetimibe combination therapies and for PCSK9 inhibitors was 40%. This was not an issue in upper-middle income countries (UMICs) and HICs (Figure 4, Panel D). One possible barrier for adequate LDL-C lowering was that 75% of the respondents in LICs reported that fixed dose statin/ezetimibe combination therapies and PCSK9 inhibitors were not registered. Respondents from LMICs reported issues with registration for all medications, but to a lesser extent. In UMICs, lack of registration of PCSK9 inhibitors was reported by only 10% of the respondents.

Regarding access to lipid lowering therapies in LICs, none of these four treatments (high- intensity statins, ezetimibe, fixed dose combinations or PCSK9 inhibitors) were reported to be universally prescribed freely in the public sector. In LICs, 50% of respondents reported that high intensity statins could be freely prescribed with 75% approximately answering that ezetimibe, fixed dose combinations of statins/ezetimibe and PCSK9 inhibitors cannot be freely prescribed. Taken together improving availability and access of high-intensity statins and ezetimibe as a basic minimum standard would alleviate a major obstacle to care in LICs. Only HICs reported that, apart from PCSK9 inhibitors, lipid lowering therapies could in most cases be prescribed freely in the public sector (Figure 4, Panel E). A similar trend emerged regarding the insurance coverage for most private patients without significant co-payment in HICs (Figure 4, Panel F). There was a strong gradient among country income categories regarding payments by patients, with the smallest patient participation in HICs. In LICs, most drugs were reported to be paid for by the patient or to have a large co-payment (apart from PCSK9 inhibitors which are largely unavailable).

The link between wealth and health and the vicious cycle has never been clearer. Health systems in LICs and LMICs must recognise the public health benefits of cholesterol lowering and consider prevention and preventive strategies as an investment rather than an expense. In this regard they must provide statins as essential medications without the burden for cardiovascular health being placed on patients who can least afford to pay for these vital therapies. Novel ways of working to share the cost of investment through multi-stakeholder engagements such as public/private partnerships, NGOs, philanthropy and other approaches may help to initiate change in LICs and LMICs which can then be sustained by member nations.

### Attainment of cholesterol goals and patient adherence to treatment plan

In the recent survey performed by the WHF, across WHO regions overall, 44% of the respondents reported that 51–75% of patients achieved LDL-C control, 28% reported that 25–50% achieved LDL-C control, and 19% that more than 75% achieved LDL-C control. By comparison, only 11% felt that less than 25% of patients achieved LDL-C control. Separating regions by income, worryingly in LICs, only 20% of the respondents reported that 51–75% of patients achieved LDL-C control, whilst 40% reported that less than 25% of patients achieved LDL-C control. This correlates with findings from a recent study which found that, in a range of LMICs, statins are used by about one in ten eligible people for the primary prevention of cardiovascular diseases and one in five eligible people for secondary prevention [68]. A limitation of these data is that it is unclear which specific goals respondents are aiming to achieve. Given the data in the rest of the world that untreated cholesterol levels are similar across regions, use of more simplistic and pragmatic approaches such as initiation of more potent statin regimens from the outset per se or initiation of fixed dose potent statin ezetimibe combinations may improve barriers in implementation. Globally, 51% of the respondents reported that 51–75% of patients adhere to their treatment plan, 25% reported that more than 75% of patients adhere to treatment, and 26% said that less than half of the patients adhere to treatment. The proportion of respondents reporting that patient adherence was less than 50% amounted to 60% in LICs and to 42% in LMICs. As discussed previously significant roadblocks remain surrounding health literacy in LICs and LMICs. Concerted efforts will be required at the level of government or health care policy to address this.

The second part of the survey focused on a series of roadblocks solutions which had been identified in the original WHF Roadmap on Cholesterol [5]. Overall, the roadblocks previously identified persist and the prior observed gradient across countries by income category remains. Notably, across all WHO income categories, the lack of awareness surrounding FH among both the general population and physicians, as well as barriers to diagnosing and effectively treating FH have not been overcome and remain the most significant barriers to managing this inherited cholesterol disorder. In LICs and LMICs, lack of access to facilities, and awareness about the importance of ASCVD risk factor screening, and adherence to treatment, among both physicians and patients, were still reported to be significant roadblocks. Conversely, in HICs, access and awareness did not apply for the majority of respondents, although implementation was far from ideal.

### Future solutions

Regarding the possible solutions to overcoming roadblocks identified in the previous Roadmap, most remained relevant. Most participants agreed that screening campaigns for dyslipidaemias and FH were needed. The ideal scenario would be for universal cholesterol screening before the age of 18 and ideally in the first decade of life, which would also offer opportunities for child parent screening which has been suggested to be cost effective [69,70]. This approach is supported by observation that among non-index cases, diagnosis of FH occurred at a younger age and CVD was far less prevalent at diagnosis [40]. Additionally, preventing obesity has to be an integral part of public health policy as it will prevent metabolic lipid disorders and diabetes both of which increase ASCVD risk.

If cholesterol measurements including Lp(a) become more readily available and costs fall, universal cholesterol screening before the age of 18 and ideally in the first decade of life may be the ideal time window not only to establish a diagnosis of an inherited lipid disorder but also to establish treatment. Patient advocacy has a key role to play with policy makers, as shown in Europe, to set the public health policies needed globally [71,72]. Where government institutions have implemented these at scale, such as in Slovenia, this can become a beacon of best practice [73]. For children, establishing a diagnosis of FH offers opportunities to initiate early and maintain favourable lifestyles when other lifestyle related CV risk factors are largely absent, potentially helping sustain behaviours into later life. As physicals signs of hypercholesterolaemia are likely to be absent in children establishing a diagnosis of FH will rely largely on LDL-C levels and thus the availability of testing. With recent advances in genomics, monogenic or polygenic contributors to cardiovascular disease could aid early decision-making.

Potential solutions for healthcare systems should include the available, scalable, low-cost genetic testing which could cover inherited dyslipidaemias (FH and high Lp(a)). For dominant and co-dominant traits this further offers an opportunity for reverse-cascade testing. Although newer treatments are expensive and may not be available in many LMICs, combinations of potent statin regimens and ezetimibe as single pill especially if initiated early through screening/case finding, may offset therapeutic costs related to case finding later in the life-course where atherosclerosis and clinical disease are much more advanced. For FH patients with characteristics suggesting higher risk of ASCVD, proposed algorithms may help guide further intensification of treatment [43]. However, for HoFH, due consideration should be given to pricing of contemporary potentially life-saving therapies inevitably required for this condition. Moreover, regulators and industry should ensure early paediatric testing of these therapies and their availability in LMICs will be needed, as it is here that the global burden of HoFH lies.

Clinical practice guidelines to prevent cardiovascular events focus on recommending pharmacologic therapy to lower LDL-C among persons with advanced atherosclerosis. Moreover, combinations of pharmacologic therapies to more intensely lower LDL-C among persons with a history of ASCVD events, are designed to be implemented in resource-rich HICs. Therefore, these guidelines may not be optimal for implementation in resource-limited LMICs. Indeed, adopting this strategy to prevent ASCVD events in LMICs could exacerbate healthcare inequities because these countries are unlikely to be able to afford expensive combinations of therapies required to achieve very low LDL-C targets, thus potentially leading to worse outcomes among persons in LMICs following an acute cardiovascular event. Instead, a more effective strategy for LMICs may be to proactively prevent ASCVD events by reducing cumulative exposure to LDL-C, the main cause of atherosclerosis. Lifetime cumulative exposure to LDL-C can be reduced by maintaining modest long-term reductions in LDL-C beginning earlier in life with diet or the combination of diet and low-dose generic LDL-C lowering therapies. Indeed, maintaining modest long-term reductions in LDL-C beginning earlier in the atherosclerotic disease process is likely to be more effective at preventing the lifetime risk of ASCVD events, and substantially less expensive, than focusing on more aggressive pharmacologic LDL-C lowering started later in life and therefore later in the atherosclerotic disease process. However, significant barriers must be overcome to effectively prevent ASCVD events by reducing cumulative exposure to the causes of atherosclerosis and other forms of injury to the arterial wall in in LMIC countries (**Box 2**).

**Box 2 Barriers to Effective Prevention of Lifetime Risk of Atherosclerotic Cardiovascular Disease by Reducing Cumulative Exposure to LDL in Low and Middle-Income Countries**

- **Screening for risk and lipid disorders in particular inherited disorders such as FH and Lp(a)** 
- **Lack of awareness of the effectiveness of preventing atherosclerotic cardiovascular disease:** There are significant knowledge gaps among physicians, policy makers, and the public about how atherosclerotic cardiovascular disease develops, and how effectively it can be prevented by reducing cumulative exposure to the causes of atherosclerosis (LDL and other apo B-containing lipoproteins) and other modifiable causes of injury to the arterial wall (elevated blood pressure, dysglycaemia of diabetes, and tobacco smoking).
- **Lack of available tools to empower effective prevention of atherosclerotic cardiovascular disease:** No currently available algorithms incorporate cumulative exposure to LDL or other modifiable causes of arterial wall injury when estimating the lifetime risk of atherosclerotic cardiovascular disease, or include causal effects to estimate the magnitude of the benefit that can be achieved by reducing lifetime cumulative exposure to LDL or other modifiable causes of arterial wall injury.
- **Lack of government policies focusing on preventing atherosclerotic cardiovascular disease:** Most countries have public health policies and programmes that specifically focus on preventing cancer. However, despite the fact that atherosclerotic cardiovascular disease is the leading cause of morbidity and mortality throughout the world, the largest source of healthcare expenditures, and is largely preventable; very few countries have specific policies focusing on reducing the lifetime risk of atherosclerotic cardiovascular disease.
- **Lack of infrastructure to enable effective prevention of atherosclerotic cardiovascular disease:** In low and middle-income countries in particular, there is a lack of infrastructure to systematically measure LDL (or other measures of atherogenic lipoproteins), track changes over time to estimate individual cumulative exposure to LDL and other causes of arterial wall injury, use this information to effectively provide guidance for how to reduce lifetime exposure to the causes of atherosclerotic cardiovascular disease, or execute on the delivery of lifestyle interventions and therapies that can effectively reduce cumulative exposure to the modifiable causes of disease to effectively prevent atherosclerotic cardiovascular disease.

Finally, simplified, easily implemented CVD guidelines, adaptation of risk profiling for specific regions and use of point of care testing was felt by survey respondents to be of value in improving implementation of best care. Furthermore, programmes including trained community health workers for risk evaluation could be of value in LMICs [74]. Shared decision making with patients could be enhanced through better health literacy. This will require the continuing need for public health awareness campaigns [75] aimed at increasing societal understanding about the causes of ASCVD, and its prevention including overcoming misinformation about the safety and efficacy of life saving treatments such as statins. Healthcare systems should think about preserving health rather than treating disease, which means considering prevention as an investment for the future rather than an expense. This will require greater availability and affordability of lipid lowering drugs, multi-professional teams and use of new technologies which are scalable to improve patient adherence like text messaging. As well as the public, continued education and dissemination of some of the key concepts about lifetime cumulative exposure to cholesterol, safety of cholesterol lowering and cholesterol lowering treatments will be needed, given the uncertainty within the present survey about limiting the number of statins to avoid misconceptions surrounding dose and cholesterol lowering efficacy as well as the potential utility of a polypill. A list of minimum requirements by country income status is shown in Table 2.

**Table 2:** Recommendations for population strategies, tests and therapies which should be available globally to manage lipids and ASCVD risk.


	LIC	LMIC	HIC

**Population strategies** (e.g., food labelling/regulation; tobacco legislation)	Y	Y	Y

**Screening** • Population strategy (e.g., all before 10/reverse cascade) • Individual Lipids (TC, HDL-C, LDL-C, TG, non-HDL-C) • Lp(a) • apo B • Imaging for risk stratification	Y Y	Y Y Y Y Y	Y Y Y Y Y

**Reshaping Healthcare Systems** e.g., Digital Solutions for data generation informing policy, disease management, decision support tools	Y	Y	Y

**Access to therapies** *General (primary or secondary prevention including HeFH and HoFH)* Generic high-intensity statins (essential medication) Generic statin-ezetimibe combination	Y Y Y	Y Y Y	Y Y Y

*Statin intolerant patients* Ezetimibe Bempedoic acid/bempedoic acid plus ezetimibe	Y	Y Y	Y Y

ASCVD or HeFH * PCSK9 Mab Inclisiran		(Based on LDL-C level and/or risk) Y Y	(Based on LDL-C level and/or risk) Y Y

*HoFH-* Apheresis Lomitapide Evinacumab	Y Y Y	Y Y Y	Y Y Y

*Icosapent ethyl ester*		Y	Y

*Fibrates* (as TG lowering therapies for prevention of pancreatitis)	Y	Y	Y


** In LIC industry and governments should reach agreement on significantly discounted costs so that that those with inherited lipid disorders are not disenfranchised.

## Selected actionable solutions

**Figure 5:**
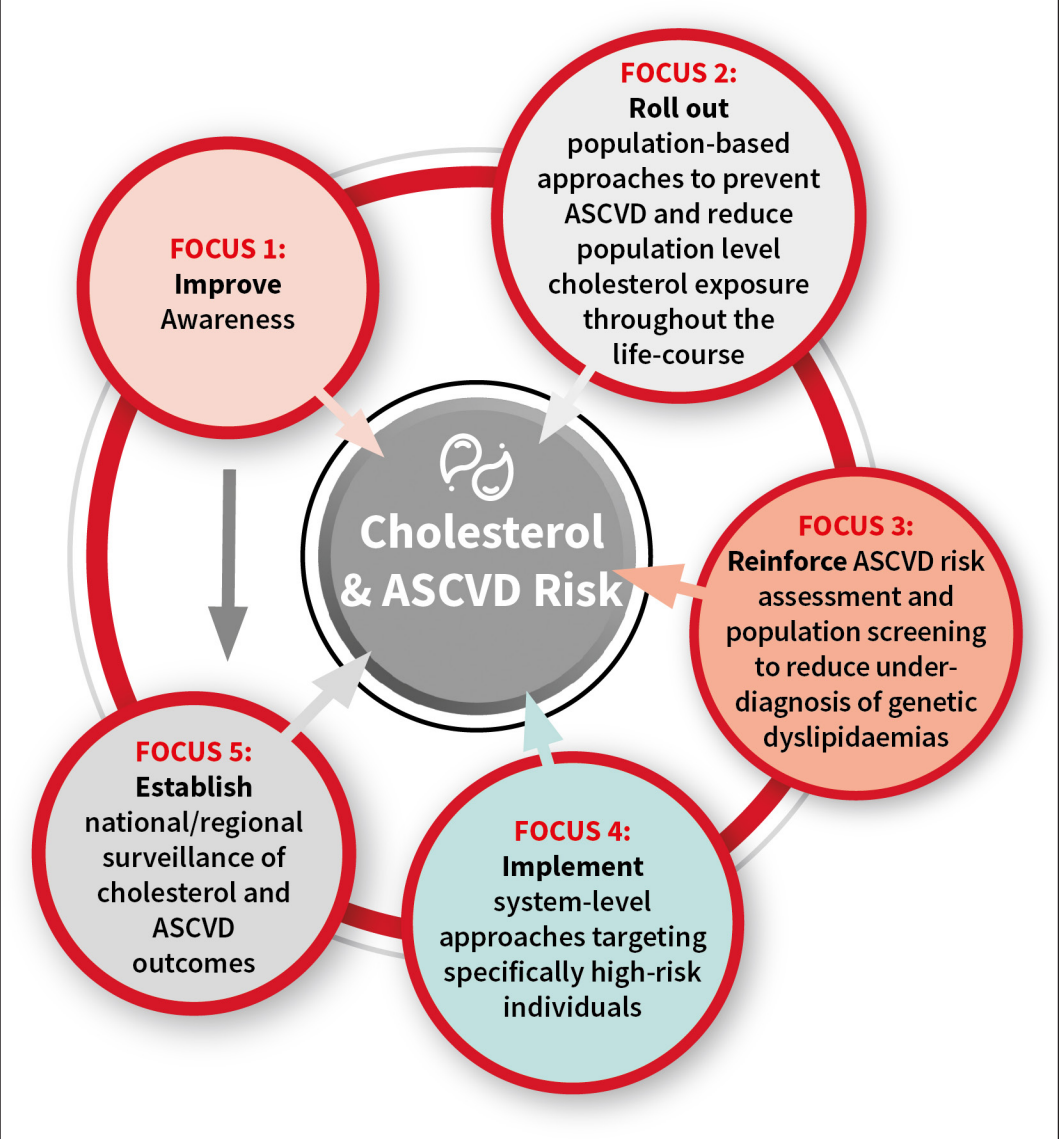
**Actionable solutions to address cholesterol control as a means to reduce ASCVD** *© World Heart Federation*.

**Figure 6:**
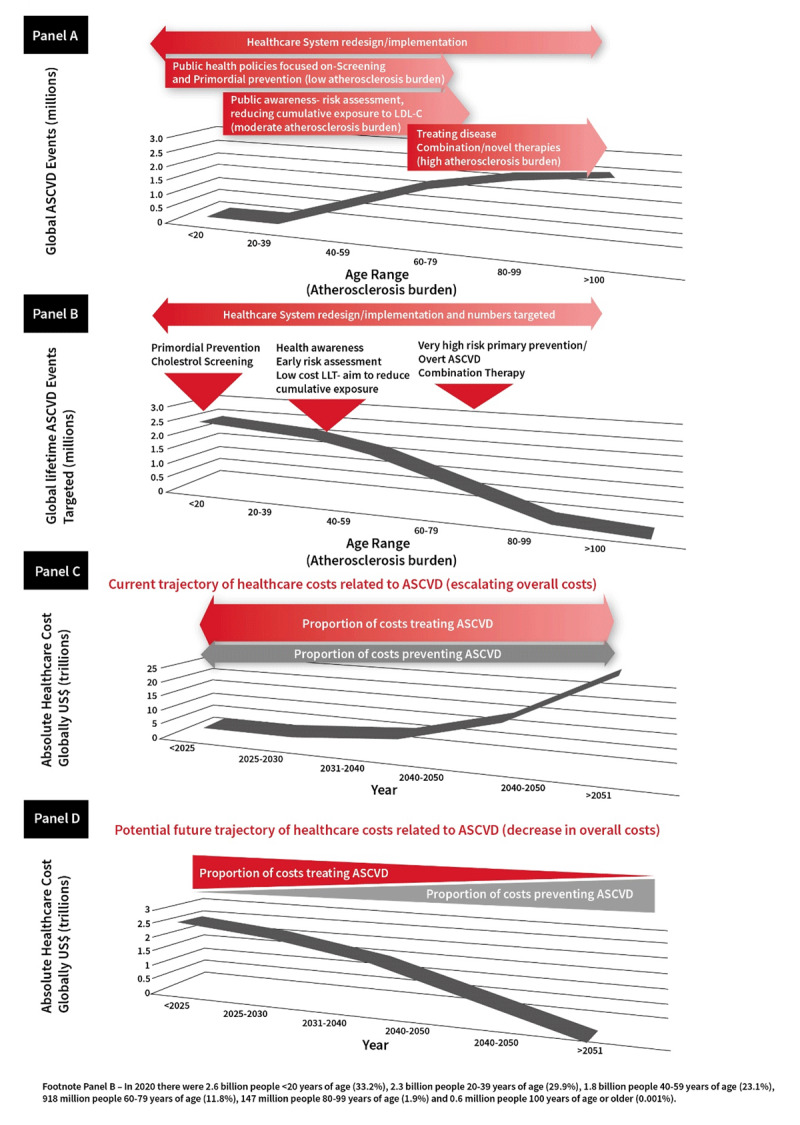
Redesigning healthcare strategies to focus on the burden of atherosclerosis throughout the life-course (panel A), with different population approaches using different ‘treatment’ strategies across different age ranges and hence the absolute numbers targeted (Panel B). Low-cost large scale forward thinking provides benefits in the longer term for those at high lifetime risk but low short-term risk and higher cost lower scale provide near time benefits in those at highest short-term risk. Panel C shows the current healthcare expenditure (exemplar) as a proportion of treating the consequences of ASCVD versus prevention of ASCVD and Panel D shows healthcare expenditure if current focus is shifted to prevention (exemplar). *© World Heart Federation*.

A series of actionable solutions to preventing ASCVD events through addressing cholesterol exposure were identified, covering 5 focus areas (Figure 5 and Table 3: Focus Areas 1–5): 1) improve awareness; 2) roll out population-based approaches to prevent ASCVD and reduce population level cholesterol exposure throughout the life-course; 3) reinforce ASCVD risk assessment and population screening to reduce under-diagnosis of genetic dyslipidaemias; 4) implement system level approaches targeting specifically high-risk individuals; 5) establish-national/regional surveillance of cholesterol and ASCVD outcomes. Moreover, understanding the importance of cumulative exposure to atherogenic lipoproteins and atherosclerosis allows the global community to redesign healthcare systems with different approaches across the life-course, which if implemented across the next decade could significantly reduce long-term healthcare costs through better preventing ASCVD (Figure 6).

**Table 3:** Five focus areas to implement actionable solutions to address cholesterol control as a means to reduce ASCVD.


FOCUS AREA 1: IMPROVE AWARENESS	FOCUS 2: ROLL OUT POPULATION-BASED APPROACHES TO PREVENT ASCVD AND REDUCE POPULATION LEVEL CHOLESTEROL EXPOSURE THROUGHOUT THE LIFE-COURSE

**Health professionals** Conduct awareness and education campaigns to ensure that health professionals: are aware of the continuous graded association between cholesterol blood levels and ASCVD risk: There is no normal cholesterol level, but adequate values based on ASCVD risk. shift focus to the impact of cumulative long-term exposure to cholesterol and risk of ASCVD [52]. change to lifetime risk estimation rather than 10-year risk only [37,77]. realise the importance of screening for elevated cholesterol including FH and Lp(a) in early life. Ensure multi-professional teams provide consistent and understandable information to patients [78]. Provide tools showing the safety of cholesterol lowering drugs and low cholesterol levels including early use of combination therapies. Adapt evidence-based risk evaluation and guideline recommendations to local needs [78,79]. **Patients and general populations [78]** Promote advocacy through greater interaction of patient organisations with healthcare professionals and policy makers. Implement a more inclusive approach in guidelines development, partnering in advocacy an policy influencing, clinical trials design and involvement in regulatory and the technical assessment process. Implement campaigns to raise awareness about the importance of screening for elevated cholesterol including FH and Lp(a). Provide educational programs/tools about lifelong exposure to cholesterol as cause of ASCVD overall. This includes differentiating between different needs among women and men, at different stage of life – childhood, adolescent, family planning phase – specially women [52]. Develop educational tools for patients as reliable resources to counterbalance misinformation, e.g., websites; social media; apps. Develop tools to increase adherence to therapy (education, apps, text messaging).	Support food reformulation efforts, in particular regarding eliminating artificial trans-fat. Follow WHO recommendations to enact a mandatory 2% limit on industrially produced TFAs in foods or to ban PHOs. The WHO’s REPLACE action package and technical support in country should be leveraged by policymakers to protect their populations from the health harms of TFAs [80]. Fully implement the WHO Framework Convention on Tobacco Control (FCTC), in particular: raising tobacco taxes, introducing comprehensive bans on tobacco advertising and sponsorship, placing large health warnings on packaging and legislating for smoke-free environments. Deliver education on healthy dietary patterns with focus on reducing consumption of ultra-processed unhealthy fat containing foods. Generate additional resources at national level, by enforcing adequate taxation policies on unhealthy commodities such as non-alcoholic beverages (e.g., sugar-sweetened beverages), tobacco and alcohol, and allocating these revenues for advancing the prevention and control of NCDs [81].

**FOCUS 3: REINFORCE ASCVD RISK ASSESSMENT AND POPULATION SCREENING TO REDUCE UNDER-DIAGNOSIS OF GENETIC DYSLIPIDAEMIAS**	**FOCUS 4: IMPLEMENT SYSTEM-LEVEL APPROACHES TARGETING SPECIFICALLY HIGH-RISK INDIVIDUALS**

**General population** Develop simplified locally-adapted guidelines [79] for whom and how to screen for ASCVD risk based on a total risk approach (consider previous ASCVD, family history and or presence of other risk factors like smoking, hypertension, diabetes, obesity, etc.) [77]. Provide risk stratification tools in easy-to-use formats (apps; websites). Focus on lifetime rather than 10-year risk estimation to guide pharmacological therapy [37,77]. Set up point-of-care testing with inexpensive and easy-to-use technologies (e.g., cholesterol test strips). **Screening for FH and Lp(a)** Develop and implement national policies for mandated screening; or adjust paediatric guidelines to promote routine universal screening and cascade screening [70]; as well as all guidelines to promote reverse cascade screening. Governments are encouraged to consider, in particular universal child-parent screening and cascade testing of first- and second-degree relatives [70,82]. Provide funds for genetic testing of FH [83]. Create care pathways for sign-posting and directing referrals to lipid clinics or tertiary facilities for patients with severe forms of FH like HoFH or HeFH patients refractory to standard lipid lowering therapies. Allocate sufficient resources for screening and diagnosis throughout the life-course, and risk stratification beginning in childhood on a fair basis, in the best interests of the child, similar to other genetic conditions.	Ensure the affordability of essential cholesterol lipid lowering medicines [84]: Ensure affordability of statin and non-statin therapies through free or subsidized drug provision, reducing taxes on pharmaceuticals. Include LLTs, esp. statins, into essential medication packages [68]. Ensure the availability of generic potent lipid lowering drugs with WHO, particularly for low- and middle-income countries. Ensure the use of generic statin+ ezetimibe single-pill combination therapies for potent cholesterol reduction, lessen therapy inertia, reduce pill burden, and cope with fear of possible adverse events of high-dose statins. Guarantee access to PCSK9 lowering therapies for cases of severe hypercholesterolemia such as FH and or established ASCVD where cholesterol levels remain well above recommended local goals. Reducing the taxation of these medications, extending patent protection could make these affordable for health care systems. Include a list of essential discounted medications, therapies for severe dyslipdaemia such as HoFH. Provide recommendations towards funding prioritization for prevention and creation of national cardiovascular health programs with the inclusion of cholesterol awareness, testing, and management, and novel therapies accessibility. Reduce complexity of local guidelines to promote first line use of generic potent cholesterol lowering combination therapies (statin + ezetimibe) for those at highest risk. Support the use of polypills (combination pill including aspirin, a beta-blocker, a statin and an ACE inhibitor) among certain high-risk groups (e.g., post-MI, diabetics [85,86]) reducing cost and promoting adherence. Support the engagement of pharmacists and non-physician health workers in patient support and counselling about drugs benefits, possible adverse events to increase adherence to drug therapy. Foster the use of novel technologies such as apps or text messaging to support patient adherence and cholesterol goal attainment. Develop and implement the use of smart decision support tools at the level of the healthcare system especially in those LMICs where EMR are available or being developed [79]. Entering into carefully selected public-private collaborations.

**FOCUS 5: ESTABLISH NATIONAL/REGIONAL SURVEILLANCE OF CHOLESTEROL AND ASCVD OUTCOMES**	

Monitor whether patients take recommended medications to reduce cholesterol and control other risk factors, and also if they renew their prescriptions. Use of apps and internet-based resources linking patients, pharmacies, and health care professionals. Creation of health care practitioner monitoring groups (pharmacists; nurses) to help patients with self-care. Monitor whether patients attain cholesterol goals as recommended. Use of apps and internet-based resources. Collect epidemiological data to provide data-driven policies and assess the impact of care -this includes collection of surveillance data on cholesterol levels, ASCVD rates and dietary patterns. Develop reliable health information systems to monitor health behaviours, risk factors, and morbidity and mortality. Implement the WHO Global Monitoring Framework. Reach agreement among governments and intergovernmental agencies upon implementing international standards. Monitor stock outages for essential medicines such as statins.	

**FOCUS 5: ESTABLISH NATIONAL/REGIONAL SURVEILLANCE OF CHOLESTEROL AND ASCVD OUTCOMES**	

Financially support the development of FH registries to quantify current practices, identify knowledge-practice gaps, publish metrics for monitoring and standardizing care, identify areas for future resource deployment, dissemination and defining best practices as well as facilitating FH awareness and screening. Developing local patient-centred approaches including patient platforms for data entry and education should be considered, ensuring optimal privacy and confidentiality. Resolving aspects related to various insurance and genetic discrimination – stop penalizing, start incentivising adherence and other good behaviours.	


## Conclusion

There is overwhelming evidence that the adverse effects of LDL-C and apo B containing lipoprotein exposure are cumulative and result in ASCVD. These are preventable by implementation of different strategies, aimed at efficiently tackling atherosclerosis at different stages throughout the human life-course. Preventive strategies should therefore be updated to implement health policy, lifestyle changes and when needed pharmacotherapies earlier with investment and a shift in focus towards early preventive strategies that preserve health and thus prevent atherosclerosis and related adverse health outcomes (ASCVD) rather than simply treat the consequences of ASCVD. This will require availability of affordable cholesterol testing, screening for inherited cholesterol disorders, a greater focus on primordial prevention, and wider availability of affordable cholesterol lowering therapies which should include potent statins as essential medications globally.

## Additional File

The additional file for this article can be found as follows:

10.5334/gh.1154.s1 Supplementary Appendix. Online supplement with additonal tables and data.
