# Supplementary material for: World Heart Federation Cholesterol Roadmap 2022
Source: Glob Heart. 2022 Oct 14;17(1):75. doi: 10.5334/gh.1154 (PMC9562775; doi:10.5334/gh.1154)
Supplement: Supplementary Appendix. — Online supplement with additonal tables and data. [file gh-17-1-1154-s1.pdf]

Supplementary Appendix

for

World Heart Federation Cholesterol Roadmap 2022

*‡Kausik K Ray, Brian A Ference, Tania Séverin, Dirk Blom, Stephen J. Nicholls, Mariko H. Shiba, Wael Almahmeed, Rodrigo Alonso, Magdalena Daccord, Marat Ezhov, Rosa Fernández Olmo, Piotr Jankowski, Fernando Lanas, Roopa Mehta, Raman Puri, Nathan D. Wong, David Wood, Dong Zhao, Samuel S. Gidding, Salim S. Virani, Donald Lloyd-Jones, Fausto Pinto, Pablo Perel, ‡Raul D. Santos

‡ **Kausik K. Ray and Raul D. Santos** are co-Chairs of the Roadmap update

* Corresponding Author

Table of Contents

eTable1 Methods for determining LDL-C.. 3

eTable 2 Distribution of LDL-C in the Framingham Offspring Study and the corresponding distribution of non-HDL-C and apo B.. 5

eTable 3: Major risk prediction algorithms for predicting 10-year ASCVD risk used globally in primary prevention... 6

eTable4 Exemplar of 10-year risk and benefits from rivaroxaban and PCSK9 lowering therapies in ASCVD patients using the SMART Risk Equation 8

eTable 5: LDL-C, apo B and non-HDL-C goals from ESC/EAS 2019 guidelines (7) 9

Pharmacological lipid lowering therapies... 10

Survey.. 13

e References.. 14

eTable1 Methods for determining LDL-C

Direct measurement of LDL-C (LDL-C_D) directly e.g. with one of the Roche, Beckman, or Siemens assays.

Homogeneous assays that directly measure LDL-C with proprietary chemicals that mask the non-LDL particles have become popular, but the methods vary in performance. As with direct HDL-C assays, the accuracy of direct LDL-C assays depends on the specificity of the masking reagents. The accuracy of these LDL-C measurements decreases with increasing TG levels (1).

Friedewald's LDL-C estimation (LDL-C_F) with the following formula: I suggest to add as table foot: In the fasting state and TG <4.5 mmol/L(400 mg/dL) and LDL-C > 1.8mmol/L (70 mg/dL)

$$\text{LDL-C}_F = \text{TC} - \text{HDL-C} - (\text{TG}/5)$$

(if reported in mg/dl or TG/2.2 for SI)

For patients with LDL-C >100 mg/dL and TG <150 mg/dL it is reasonable to use the Friedewald formula. However, for those with TG 150-400 mg/dL the Friedewald formula for LDL-C estimation is less accurate. The Martin/Hopkins method is recommended for LDL-C estimation throughout the range of LDL-C levels and up to TG levels of 399 mg/dL (1). For TG levels >400 mg/dL LDL-C estimating equations are currently not recommended and newer methods are being evaluated. Further addressing TG levels >400 mg/dL situation further, there may be some benefit of an extended version of Martin Hopkins approach over Sampson (2).

Sampson's LDL-C estimation (LDL-C_S) using the least squares formula:

$$\text{LDL-C}_S = \frac{\text{TC}}{0.948} - \frac{\text{HDL-C}}{0.971} - \left(\frac{\text{TG}}{8.56} + \frac{\text{TG} \times \text{non-HDL-C}}{2140} - \frac{\text{TG}^2}{16100}\right) - 9.44$$

Martin-Hopkins LDL-C estimations were calculated using the formula :

$$\text{LDL-C}_M = \text{TC} - \text{HDL-C} - (\text{TG}/\zeta)$$

In this formula, ζ is an adjustable factor and was calculated using the median TG/VLDL-C ratio, which takes into account the sublevels of TG and non-HDL-C levels (3, 4).

eTable 2 Distribution of LDL-C in the Framingham Offspring Study and the corresponding distribution of non-HDL-C and apo B

This image cannot currently be displayed.

LDL-P- LDL lipoprotein particle concentration

eTable 3: Major risk prediction algorithms for predicting 10-year ASCVD risk used globally in primary prevention

Name	Pool Cohort equation	Q Risk 3	SCORE2	SCORE 2 OP
Derivation Country/ Region<br><br>Citation	USA<br><br>Performance of the Pooled Cohort Equations to Estimate Atherosclerotic Cardiovascular Disease Risk by Body Mass Index (5)<br><br>2018	UK<br><br>Cardiovascular Risk Assessment (6)<br><br>Q Risk 3 Calculator(7)<br><br>2018	Europe<br><br>SCORE2 Risk prediction algorithms: new models to estimate 10-year risk of cardiovascular disease in Europe(8)<br><br>2021	Europe<br><br>SCORE2-OP risk prediction algorithms: estimating incident cardiovascular event risk in older persons in four geographical risk regions(9)<br><br>2021
Variables included	Are age, sex, race (ie, White, Black, or other), smoking status, systolic blood pressure, hypertension treatment status, diabetes status, and total and high-density lipoprotein (HDL) cholesterol levels.	Age (25-84), sex., ethnicity, smoking status, diabetes, angina or heart attack in a first-degree relative <60, CKD stage 3, 4 or 5, atrial fibrillation, existing treatment with blood pressure agent, postcode (geographical measure of deprivation), migraines, rheumatoid arthritis, systemic lupus erythematosus (SLE), severe mental illness, atypical antipsychotics, regular steroid tablets, diagnosed erectile dysfunction, BMI, systolic blood pressure, total and HDL cholesterol and self-assigned ethnicity	Age (40-69), sex, smoking status, systolic blood pressure, non-HDL-C levels.<br><br>The Score-2 risk prediction algorithm is available for four distinct European risk regions.	Age (>70), sex, smoking status, systolic blood pressure, non-HDL-C levels.<br><br>The Score-OP2 risk prediction algorithm is available for four distinct European risk regions
Outcomes	Fatal stroke, nonfatal stroke, fatal coronary heart disease, and nonfatal myocardial infarction.	estimates the 10-year risk of CVD, including MI, CHD, stroke, and transient ischemic attack	Fatal stroke, nonfatal stroke, fatal coronary heart disease, and nonfatal myocardial infarction.	Fatal stroke, nonfatal stroke, fatal coronary heart disease, and nonfatal myocardial infarction.
Treatment thresholds for Lipid	Adults aged 40-75 with LDL-C 70-189 and without diabetes: Borderline risk (5<7.5%),	People who have a 10% or greater risk of developing CVD within the next 10 years or FH	Age dependent threshold for statin therapy	A statin may be considered for primary prevention in older people if at high risk or above.

Lowering Therapies	intermediate risk (7.5<20%) and high risk >=20%.<br><br>High risk (≥20%) initiate high intensity statin therapy<br><br>Adults aged 40-75 with LDL-C ≥ 190: Initiate high intensity statin<br><br>Adults aged 40-75 with LDL-C 70-189 and with diabetes<br><br>Risk <7.5% Initiate moderate-intensity statin<br><br>Risk ≥ 7.5% or risk modifiers: Consider high intensity statin			
/ Recommendations	In those with LDL-C 70–189 mg/dL, without diabetes mellitus, and 10-year ASCVD risk ≥20%; a goal of ≥50% reduction of LDL-C with a high intensity or maximally tolerated statin<br><br>In those with diabetes mellitus, 40–75 years of age, LDL-C 70–189 mg/dl (1.8–4.9 mmol/L), moderate intensity statin to lower LDL-C 30%–49%	10 year risk > 10%<br><br>Links into NICE 2014<br><br>ATV 20mg or Rosuvastatin 10mg, uptitrate to achieve >40% lowering of non-HDL-C<br><br>No target	< 50 years threshold for treatment is >=7.5% and 50-69 is >=10% for initiating statins<br><br>**Low risk**: A goal of <3·0 mmol/L (<116 mg/dL)<br><br>**Moderate risk**: A goal of <2·6 mmol/L (<100 mg/dL)<br><br>**High risk**: A therapeutic regimen that achieves ≥50% LDL-C reduction from baseline and an LDL-C goal of <1·8 mmol/L (<70 mg/dL)<br><br>**Very high risk**: A therapeutic regimen that achieves ≥50% LDL-C reduction from baseline and an LDL-C goal of <1·4 mmol/L (<55 mg/dL)	For age >=70 years threshold for treatment is >=15% for initiating statins

eTable4 Exemplar of 10-year risk and benefits from rivaroxaban and PCSK9 lowering therapies in ASCVD patients using the SMART Risk Equation

Patient	1	2	3	4	5	6	7	8	9	10	11	12	13
non-HDL cholesterol (mmol/L)	2.79	3.26	2.95	3.07	2.89	2.61	2.79	3.20	3.49	3.49	3.49	3.49	3.49
HDL cholesterol (mmol/L)	0.517	0.517	0.491	0.698	0.646	0.698	0.698	0.517	0.749	0.646	0.465	0.517	0.646
Total cholesterol (mmol/L)	3.31	3.77	3.44	3.77	3.54	3.31	3.49	3.72	4.24	4.13	3.95	4.01	4.13
Age (years)	63.6	57.0	62.6	46.1	74.7	71.0	72.1	72.0	73.4	62.3	69.0	57.1	77.0
Sex	M	M	F	M	F	F	F	M	F	M	M	F	M
Current smoking status	No	Yes	No	Yes	No	Yes	Yes	No	No	No	No	No	No
Systolic BP (mm Hg)	114	150	140	130	165	148	150	160	170	156	160	145	110
Diabetes Mellitus	Yes	No	No	No	No	No	No	No	No	No	No	No	No
Coronary Heart Disease	Yes	Yes	Yes	No	No	No	Yes	Yes	Yes	No	No	Yes	Yes
Cerebrovascular Disease	No	No	No	Yes	No	No	No	No	No	Yes	No	No	Yes
Abdominal Aortic Aneurysm	No	No	No	Yes	No	No	No	No	No	No	No	No	No
Peripheral Vascular Disease	No	No	No	No	Yes	Yes	No	No	No	No	Yes	No	No
Years since ASCVD	-	-	2.2	-	11.8	-	12.6	14.5	23.9	4.5	7.5	-	21.5
eGFR (ml/min/1.73m)	67.7	71.0	54.1	71.5	78.6	48.4	59.9	75.3	30.9	85.7	54.2	53.1	91.1
hsCRP (mg/L) imputed	2.0	2.3	2.1	4.5	3.4	3.4	2.7	2.5	2.7	2.0	2.4	2.5	2.5
SMART 10 -year predicted baseline risk (%)	20.0	20.0	20.0	20.0	40.0	40.0	40.0	40.0	75.7	23.8	45.4	18.1	61.7
Addition of rivaroxaban													
Predicted 10-year risk and 95 % CI	15.2 (13.2-17.2)	15.2 (13.2-17.2)	15.2 (13.2-17.2)	15.2 (13.2-17.2)	30.4 (26.4-34.4)	30.4 (26.4-34.4)	30.4 (26.4-34.4)	30.4 (26.4-34.4)	57.5 (50.0-65.1)	18.1 (15.7-20.5)	34.5 (30.0-39.0)	13.8 (11.9-15.6)	46.9 (40.7-53.1)
Absolute risk reduction (%)	4.8	4.8	4.8	4.8	9.6	9.6	9.6	9.6	18.2	5.7	10.9	4.3	14.8
Addition of a PCSK9 MAb													
Estimated reduction in non -HDL cholesterol (mmol/L)	1.40	1.63	1.47	1.54	1.45	1.30	1.40	1.60	1.74	1.74	1.74	1.74	1.74
Predicted 10-year risk and 95% CI	15.5 (15.0-15.9)	14.8 (14.3-15.3)	15.2 (14.8-15.7)	15.1 (14.6-15.5)	30.6 (29.7-31.5)	31.4 (30.6-32.2)	30.9 (30.0-31.7)	29.7 (28.8-30.7)	54.8 (52.9-56.7)	17.2 (16.6-17.8)	32.9 (32.7-34.0)	13.1 (12.7-13.6)	44.7 (43.1-46.2)
Absolute risk reduction (%)	4.6	5.2	4.8	5.0	9.4	8.6	9.1	10.3	20.9	6.6	12.5	5.0	17.0

Adapted from McKay A EJPC 2021 (10)

eTable 5: LDL-C, apo B and non-HDL-C goals from ESC/EAS 2019 guidelines (11)

Risk Category	LDL-C goals	Apo B goals	non-HDL-C goals
**Low**	< 116 mg/dl (<3.0 mmol/L	None	none
**Moderate**	< 100 mg/dl (<2.6 mmol/L)	< 100 mg/dl	< 130 mg/dl (<3.4 mmol/L)
**High**	Reduce by ≥50% from baseline AND to < 70 mg/dl (<1.8 mmol/L)	< 80 mg/dl	< 100 mg/dl (<2.6 mmol/L)
**Very high**	Reduce by ≥50% from baseline AND to < 55 mg/dl (<1.4 mmol/L)	< 65 mg/dl	< 85 mg/dl (<2.2 mmol/L)
**Very high with recurrent ASCVD events**	< 40 mg/dl (<1.0 mmol/L)	< 55 mg/dl	< 70 mg/dl (<1.8 mmol/L)

Pharmacological lipid lowering therapies

Oral Therapies

Statins

Statin therapy is recommended as first-line pharmacological treatment in patients with sufficiently elevated cardiovascular risk. Statins inhibit the HMG-Co reductase, a key enzyme in the cholesterol synthesis pathway. This leads to a decrease in intracytosolic cholesterol, and compensatory up-regulation in *LDLR* resulting in more available LDL-R on the hepatocyte surface which increases LDL-C/apo B lipoprotein clearance. LDL-C lowering is proportional to dosing/intensity of treatment with both higher and more potent regimens achieving approximately 50% or higher LDL-C lowering.

Ezetimibe

Ezetimibe, an inhibitor of intestinal cholesterol absorption, binds to the Niemann-Pick C1-Like 1-(NPC1L1)-cholesterol complex and prevents its endocytosis by the enterocyte. This decreases the intestinal cholesterol uptake by 54% and consequently circulating LDL-C levels. On average LDL-C reductions of between 20-25% are generally expected with the 10mg dose. Lowering LDL-C through ezetimibe as an add on to statins has been shown to reduce cardiovascular events proportional to the absolute reduction in LDL-C and duration of therapy (12) as well as monotherapy (13).

Bempedoic acid

Bempedoic acid is a small molecule inhibiting a key enzyme in the cholesterol syntheses pathway (ACLY) upstream of the target for statins (HMG-CoA reductase). As a prodrug administered daily at 180mg, it requires intracellular activation in the liver by very-long-chain acyl-CoA synthetase, which is largely absent in skeletal muscle, thus attenuating the likelihood of the most common of statin-related adverse event, namely muscle aches. As bempedoic acid works in the same pathway as statins, its efficacy depending upon the intensity of background statin therapy. Among patients on high or moderate intensity statins LDL-C reductions of ~ 18%(14) with greater reduction of up to ~ 21-28% among patients on little or no statin therapy (15). A fixed-dose combination pill with ezetimibe is available, reducing medication /pill burden and providing LDL-C reductions of 38% (16), with the possibility of 3 drug single combination pills containing low dose statins, bempedoic acid and ezetimibe allowing reductions in LDL-C of 61% (17).Though cardiovascular outcome data are awaited (expected 2023), mendelian randomisation studies, suggest that LDL-C reductions achieved through genetic proxies for bempedoic acid activity should show benefits proportional to absolute reductions in LDL-C similar to statins, ezetimibe and PCSK9 lowering therapies(18).

Lomitapide

Lomitapide is a small molecule requiring daily dosing used exclusively in patients with HoFH. It is an inhibitor of the microsomal triglyceride transfer protein, which reduces the production of VLDL leading to 38-50% lowering of circulating LDL-C levels. However, this leads to accumulation of hepatic fat the long-term effects of which are uncertain (19). This is used in homozygous FH where LDL-R activity is severely impaired or absent through a reduction in VLDL assembly in the liver.

Injectable Therapies

Monoclonal Antibodies

Monoclonal antibodies are pharmaceutically engineered immunologloblulins administered either as sub-cutaneous or intravenous injections which recognise specific regions of circulating proteins. When these monoclonal antibodies bind to circulating proteins they reduce the amount of free target protein available in the circulation to exert biological effects. To date therapies have been developed against two key proteins, PCSK9 and ANGPTL3.

Monoclonals against PCSK9

There are two approved monoclonal antibodies against PCSK9 (evolcoumab and alirocumab). These therapies reduce LDL-C by between 50-60%, irrespective of the type of background oral lipid therapy (statins, or ezetimibe or both) and can be used as monotherapy among those unable to tolerate oral lipid lowering therapies. These therapies have been shown to reduce cardiovascular events in two large outcome trials(20, 21) as well as regress atherosclerosis and improve characteristics consistent with improving plaque stabilization when used in combination with oral LLTs and extremely low levels of LDL-C are achieved (22-24) . As these therapies bind all circulating PCSK9, but do not stop the main source of production (the liver), they need to self-administered every two weeks in order to provide sustained reductions in LDL-C. These therapies have been shown to produce similar reductions in LDL-C in patients with HeFH irrespective of genotype (25, 26). As the effects of these therapies rely on residual LDL-R function the effect in HoFH is more variable. Reductions of between 24-44% have been observed except for so called null/null cases where there is <2% LDL receptor activity and there are two affected alleles (25, 27).

Monoclonals against ANGPTL3

Currently there is only one approved monoclonal antibody against ANGPTL3. This is approved in patients in HoFH where LDL-C reductions of ~49% are observed (28). As LDL-C lowering is achieved through clearance via LDL-R independent pathways, with consistent reductions are observed irrespective of genotype. Importantly, these reductions are observed irrespective of background lipid-lowering therapies (mAbs against PCSK9, lomitapide and lipid apheresis). This treatment however is administered monthly via IV infusion over approximately 2hrs and therefore its use requires infrastructure which may limit its more generalised use as well.

RNA based therapies

siRNA

Inclisiran

Inclisiran is the first in a class of therapies called small interference RNA (siRNA) therapies. This consists of two RNA strands, one the anti-sense strand and the other the sense or passenger strand, conjugated to triantennary N-acetylgalactosamine (GalNAc) carbohydrates, allowing liver specific uptake. In the cytoplasm the RNA strands separate with the retention of the anti-sense strand in the endogenous RNA induced silencing complex (RISC), which directs targeted binding of the inclisiran antisense strand to PCSK9 m-RNA, triggering catalytic cleavage of multiple m-RNA strands. Hence, PCSK9 protein translation is inhibited through PCSK9 mRNA degradation. As approximately 80% of circulating PCSK9 in blood arises from the liver, inhibiting hepatic PCSK9 synthesis produces reductions in circulating LDL-C by approximately 52% over 1 year with two doses, allowing the convenience of infrequent dosing (29). Inclisiran is

approved for LDL-C reduction and is being evaluated in cardiovascular outcome trials. Inclisiran is recommended to be administered by a healthcare professional, which is a relevant consideration for its use, but a treatment administered by a healthcare professional could help overcome one of the major challenge that has held back patient self-management namely long-term non-adherence to medication.

Combination therapies

An inevitable consequence of the lowering of recommended cholesterol goals around the world is the need for use of multi-drug combinations targeting different pathways in cholesterol regulation. This is not readily appreciated, and the current step wise approach often results in clinical inertia, which in turn results in inadequate control of cholesterol, remonstrated almost serially by recurrent surveys and registries. Oral combinations of two therapies are already available in the form of a single pill; statins (at various doses) and ezetimibe and bempedoic acid plus ezetimibe which may reduce pill burden and help long-term adherence.

Triglycerides

To date no trial in the post statin era has demonstrated that the magnitude of TG lowering relates to reductions in risk of CV events. That said there is clear evidence that those with elevated TG are at higher CV risk. Most guidelines recommend more intensive non-HDL-C or apoB lowering for these patients as LDL-C levels to not account for the additional cholesterol carried in triglyceride containing apoB lipoproteins (11, 30). However, among patients with high TG the use of eisocapentethyl resulted in significant reductions in CV events despite only modest TG lowering suggesting beneficial pathways independent of lipid lowering. However, concerns about the comparator, have meant that the magnitude of the benefit is uncertain. That said some guidelines recommend high dose eicosapentethyl as a potential option to reduce CV risk among patients with high TG and on statin therapy. Although fibrates which are PPAR alpha agonists lower TG and used to prevent pancreatitis there is little contemporary data to support their general routine use for CVD prevention over other medications. Volanesorsen is an early generation ASO targeting ApoC-III which is available for familial chylonomicronaemia syndrome in those with high TG levels to prevent pancreatitis (31). There are no data to support targeting ApoC-III with this therapy as a means to reduce CVD risk.

Future therapies

Combinations of three small molecule combination therapies have been tested (low dose statin plus bempedoic acid and ezetimibe) which lower LDL-C by approximately 65%. This approach may offer potent LDL-C lowering regimens with a non-parental therapeutic approach, without the need for polypharmacy or potential real or perceived tolerability issues that may limit use of the highest doses of statins (17). This combination is currently commercially unavailable. The therapeutic potential of CETP inhibitors as a means to reduce the risk of CVD, was finally demonstrated with anacetrapib (32). However, this drug is retained in adipose tissue, thus prolonging its duration of action. This raised concerns about potential long-term safety and ultimately anacetrapib was not further developed. The CV benefit in the REVEAL trial was proportional to the absolute magnitude of apo B lowering and unrelated to the magnitude of HDL-C raising. This has led to emerging interest in this class with obicetrapib entering phase 3 trials in 2022. This may offer another oral, potentially lower cost therapeutic option, alone or as a single pill combination with ezetimibe. Other small molecules that have shown promise are therapies targeting PCSK9 (Phase1).

Another approach for targeting hepatic proteins regulating lipid metabolism at the level of mRNA is through anti-sense oligonucleotides (ASO). Whilst siRNAs are double-stranded and work in the cytoplasm, ASOs consist of a single-strand of RNA, which directly binds to mRNA inside the nucleus, triggering catalytic breakdown through RNase H resulting in the inhibition of translation. However, even with GALNAc conjugation this approach appears to require monthly dosing. ASO based therapies which lower Lp(a) by ~90% are being evaluated in CV outcome trials (33). The first ASO targeting ANGPTL3 was discontinued at phase 2B stage as TG, and apoB lowering was felt to be modest with dose dependent increases in liver function abnormities and accumulation of hepatic fat (34). Two siRNA-based therapies are in advanced stage of development for Lp(a) lowering. As compared to ASO based approaches for Lp(a) lowering this approach may only require twice yearly dosing (35, 36). In the future base editing or gene editing of PCSK9 and other targets using CRISPR technologies may offer a once in a lifetime therapy to reduce long-term exposure to LDL-C although much remains to be clarified about safety(37). Finally vaccination against PCSK9 which is entering Phase 1 may offer low cost reductions in long-term exposure to LDL-C and atherogenic lipoproteins. Existing vaccines for other diseases have a low cost of goods thus a vaccine which is safe that provides endogenous antibodies against PCSK9, through vaccination could lower LDL-C and thus potentially scalable and deliverable through existing networks in LMIC.

Survey

Overall, most respondents recognize that they follow an ASCVD prevention guideline to manage LDL-C with 62.38% following the ESC/EAS guidelines followed by ACC/AHA guidelines (44.55%) and local guidelines (34.58%). As a consequence of this the most popular tool for risk stratification in primary prevention of ASCVD among was the ESC score charts (43.56%) followed by ACC pooled cohort equation (24.75%) and, Framingham (19.8%) scores. A small number of respondents used the WHO Risk Chart (5.94%), QRisk or JBS3 (4.95%) and other non-specified tools (15%). Only 4.95% did not use a risk stratification tool. Here again, practices differ among regions. Respondents from the WHO European region mostly rely on ESC score charts. Respondents from the WHO Eastern Mediterranean region on ACC/AHA pool cohort equation, with a more hybrid picture in other regions. Of importance in all WHO income regions the ESC Score charts were the most used. In our view the greatest roadblock for these findings is the absence of validation of these risk tools for regions that they were not primarily aimed (e.g., ESC score and pool risk equations were developed specifically for Europe and the USA respectively and not tested in Africa or Latin America for example). This may bring uncertainty about the accuracy of these risk tools that may negatively influence on clinical practice. Moreover, SCORE predicts case fatalities which underestimates risk and hence delays treatment especially in younger people. This could be improved using the new SCORE 2 charts for high or very high-risk regions or equivalent.

e References

1. Wilson PW, Jacobson TA, Martin SS, Jackson EJ, Le N-A, Davidson MH, et al. Lipid Measurements in the Management of Cardiovascular Diseases: Practical Recommendations - a scientific statement from the national lipid association writing group. 2022.
2. Sajja A, Park J, Sathiyakumar V, Varghese B, Pallazola VA, Marvel FA, et al. Comparison of Methods to Estimate Low-Density Lipoprotein Cholesterol in Patients With High Triglyceride Levels. JAMA Netw Open. 2021;4(10):e2128817.
3. Martin SS, Blaha MJ, Elshazly MB, Toth PP, Kwiterovich PO, Blumenthal RS, et al. Comparison of a novel method vs the Friedewald equation for estimating low-density lipoprotein cholesterol levels from the standard lipid profile. Jama. 2013;310(19):2061-8.
4. Sampson M, Ling C, Sun Q, Harb R, Ashmaig M, Warnick R, et al. A New Equation for Calculation of Low-Density Lipoprotein Cholesterol in Patients With Normolipidemia and/or Hypertriglyceridemia. JAMA Cardiol. 2020;5(5):540-8.
5. Khera R, Pandey A, Ayers CR, Carnethon MR, Greenland P, Ndumele CE, et al. Performance of the Pooled Cohort Equations to Estimate Atherosclerotic Cardiovascular Disease Risk by Body Mass Index. JAMA Network Open. 2020;3(10):e2023242-e.
6. Jarvis S. Cardiovascular Risk Assessment 2020 [Available from: https://patient.info/doctor/cardiovascular-risk-assessment#.
7. ClinRisk. QRISK®3-2018 risk calculator 2018 [Available from: https://qrisk.org/three/.
8. SCORE2 working group ESC Cardiovascular risk collaboration. SCORE2 risk prediction algorithms: new models to estimate 10-year risk of cardiovascular disease in Europe. European Heart Journal. 2021;42(25):2439-54.
9. SCORE2-OP working group ESC Cardiovascular risk collaboration. SCORE2-OP risk prediction algorithms: estimating incident cardiovascular event risk in older persons in four geographical risk regions. European Heart Journal. 2021;42(25):2455-67.
10. McKay AJ, Gunn LH, Ference BA, Dorresteijn JAN, Berkelmans GFN, Visseren FLJ, et al. Is the SMART risk prediction model ready for real-world implementation? A validation study in a routine care setting of approximately 380 000 individuals. Eur J Prev Cardiol. 2021.
11. ESC Committee for Practice Guidelines (CPG), Societies ENC. 2019 ESC/EAS guidelines for the management of dyslipidaemias: Lipid modification to reduce cardiovascular risk. Atherosclerosis. 2019;290:140-205.
12. Cannon CP, Blazing MA, Giugliano RP, McCagg A, White JA, Theroux P, et al. Ezetimibe Added to Statin Therapy after Acute Coronary Syndromes. New England Journal of Medicine. 2015;372(25):2387-97.
13. Ouchi Y, Sasaki J, Arai H, Yokote K, Harada K, Katayama Y, et al. Ezetimibe Lipid-Lowering Trial on Prevention of Atherosclerotic Cardiovascular Disease in 75 or Older (EWTOPIA 75): A Randomized, Controlled Trial. Circulation. 2019;140(12):992-1003.
14. Ray KK, Bays HE, Catapano AL, Lalwani ND, Bloedon LT, Sterling LR, et al. Safety and Efficacy of Bempedoic Acid to Reduce LDL Cholesterol. N Engl J Med. 2019;380(11):1022-32.
15. Banach M, Duell PB, Gotto AM, Jr., Laufs U, Leiter LA, Mancini GBJ, et al. Association of Bempedoic Acid Administration With Atherogenic Lipid Levels in Phase 3 Randomized Clinical Trials of Patients With Hypercholesterolemia. JAMA Cardiol. 2020;5(10):1124-35.

16. Ballantyne CM, Laufs U, Ray KK, Leiter LA, Bays HE, Goldberg AC, et al. Bempedoic acid plus ezetimibe fixed-dose combination in patients with hypercholesterolemia and high CVD risk treated with maximally tolerated statin therapy. Eur J Prev Cardiol. 2020;27(6):593-603.
17. Rubino J, MacDougall DE, Sterling LR, Hanselman JC, Nicholls SJ. Combination of bempedoic acid, ezetimibe, and atorvastatin in patients with hypercholesterolemia: A randomized clinical trial. Atherosclerosis. 2021;320:122-8.
18. Ference BA, Ray KK, Catapano AL, Ference TB, Burgess S, Neff DR, et al. Mendelian Randomization Study of ACLY and Cardiovascular Disease. N Engl J Med. 2019;380(11):1033-42.
19. Brandts J, Ray KK. Familial Hypercholesterolemia: JACC Focus Seminar 4/4. J Am Coll Cardiol. 2021;78(18):1831-43.
20. Sabatine MS, Giugliano RP, Keech AC, Honarpour N, Wiviott SD, Murphy SA, et al. Evolocumab and Clinical Outcomes in Patients with Cardiovascular Disease. N Engl J Med. 2017;376(18):1713-22.
21. Schwartz GG, Steg PG, Szarek M, Bhatt DL, Bittner VA, Diaz R, et al. Alirocumab and Cardiovascular Outcomes after Acute Coronary Syndrome. N Engl J Med. 2018;379(22):2097-107.
22. Nicholls SJ, Nissen SE, Prati F, Windecker S, Kataoka Y, Puri R, et al. Assessing the impact of PCSK9 inhibition on coronary plaque phenotype with optical coherence tomography: rationale and design of the randomized, placebo-controlled HUYGENS study. Cardiovasc Diagn Ther. 2021;11(1):120-9.
23. Nicholls SJ, Puri R, Anderson T, Ballantyne CM, Cho L, Kastelein JJ, et al. Effect of Evolocumab on Progression of Coronary Disease in Statin-Treated Patients: The GLAGOV Randomized Clinical Trial. Jama. 2016;316(22):2373-84.
24. Räber L, Ueki Y, Otsuka T, Losdat S, Häner JD, Lonborg J, et al. Effect of Alirocumab Added to High-Intensity Statin Therapy on Coronary Atherosclerosis in Patients With Acute Myocardial Infarction: The PACMAN-AMI Randomized Clinical Trial. Jama. 2022;327(18):1771-81.
25. Raal FJ, Honarpour N, Blom DJ, Hovingh GK, Xu F, Scott R, et al. Inhibition of PCSK9 with evolocumab in homozygous familial hypercholesterolaemia (TESLA Part B): a randomised, double-blind, placebo-controlled trial. Lancet. 2015;385(9965):341-50.
26. Brandts J, Dharmayat KI, Vallejo-Vaz AJ, Azar Sharabiani MT, Jones R, Kastelein JJP, et al. A meta-analysis of medications directed against PCSK9 in familial hypercholesterolemia. Atherosclerosis. 2021;325:46-56.
27. Santos RD, Stein EA, Hovingh GK, Blom DJ, Soran H, Watts GF, et al. Long-Term Evolocumab in Patients With Familial Hypercholesterolemia. J Am Coll Cardiol. 2020;75(6):565-74.
28. Raal FJ, Rosenson RS, Reeskamp LF, Hovingh GK, Kastelein JJP, Rubba P, et al. Evinacumab for Homozygous Familial Hypercholesterolemia. N Engl J Med. 2020;383(8):711-20.
29. Wright RS, Ray KK, Raal FJ, Kallend DG, Jaros M, Koenig W, et al. Pooled Patient-Level Analysis of Inclisiran Trials in Patients With Familial Hypercholesterolemia or Atherosclerosis. J Am Coll Cardiol. 2021;77(9):1182-93.
30. Grundy SM, Stone NJ, Bailey AL, Beam C, Birtcher KK, Blumenthal RS, et al. 2018 AHA/ACC/AACVPR/AAPA/ABC/ACPM/ADA/AGS/APhA/ASPC/NLA/PCNA Guideline on the Management of Blood Cholesterol: A Report of the American College of Cardiology/American Heart Association Task Force on Clinical Practice Guidelines. Circulation. 2019;139(25):e1082-e143.
31. Witztum JL, Gaudet D, Freedman SD, Alexander VJ, Digenio A, Williams KR, et al. Volanesorsen and Triglyceride Levels in Familial Chylomicronemia Syndrome. N Engl J Med. 2019;381(6):531-42.

32. Bowman L, Hopewell JC, Chen F, Wallendszus K, Stevens W, Collins R, et al. Effects of Anacetrapib in Patients with Atherosclerotic Vascular Disease. N Engl J Med. 2017;377(13):1217-27.
33. Tsimikas S, Karwatowska-Prokopczuk E, Gouni-Berthold I, Tardif JC, Baum SJ, Steinhagen-Thiessen E, et al. Lipoprotein(a) Reduction in Persons with Cardiovascular Disease. N Engl J Med. 2020;382(3):244-55.
34. Bergmark BA, Marston NA, Bramson CR, Curto M, Ramos V, Jevne A, et al. Effect of Vupanorsen on Non-High-Density Lipoprotein Cholesterol Levels in Statin-Treated Patients With Elevated Cholesterol: TRANSLATE-TIMI 70. Circulation. 2022;145(18):1377-86.
35. Koren MJ, Moriarty PM, Baum SJ, Neutel J, Hernandez-Illas M, Weintraub HS, et al. Preclinical development and phase 1 trial of a novel siRNA targeting lipoprotein(a). Nat Med. 2022;28(1):96-103.
36. Nissen SE, Wolski K, Balog C, Swerdlow DI, Scrimgeour AC, Rambaran C, et al. Single Ascending Dose Study of a Short Interfering RNA Targeting Lipoprotein(a) Production in Individuals With Elevated Plasma Lipoprotein(a) Levels. Jama. 2022;327(17):1679-87.
37. Rifai MA, Ballantyne CM. PCSK9-targeted therapies: present and future approaches. Nat Rev Cardiol. 2021;18(12):805-6.
